# Time-of-Day Immunotherapy Administration and Outcomes in Advanced Cancers: A Systematic Review and Meta-Analysis

**DOI:** 10.1001/jamanetworkopen.2026.10815

**Published:** 2026-05-05

**Authors:** Shota Inoue, Ichiro Tsuboi, Marcin Miszczyk, Keiichiro Miyajima, Navid Roessler, Ahmed R. Alfarhan, Satoshi Katayama, Pierre I. Karakiewicz, Motoo Araki, Shahrokh F. Shariat

**Affiliations:** 1 Department of Urology, Comprehensive Cancer Center, Medical University of Vienna, Vienna, Austria; 2 Division of Anatomy, Medical University of Vienna, Vienna, Austria; 3 Department of Urology, Okayama University Graduate School of Medicine, Dentistry and Pharmaceutical Sciences, Okayama, Japan; 4 Collegium Medicum-Faculty of Medicine, WSB University, Dąbrowa Górnicza, Poland; 5 Department of Urology, The Jikei University School of Medicine, Tokyo, Japan; 6 Department of Urology, University Medical Center Hamburg-Eppendorf, Hamburg, Germany; 7 Department of Urology, Prince Saud Bin Jalawi Hospital, Al Ahsa Health Cluster, Al Ahsa, Saudi Arabia; 8 Cancer Prognostics and Health Outcomes Unit, Division of Urology, University of Montréal Health Center, Montréal, Québec, Canada; 9 Department of Urology, Weill Cornell Medical College, New York, New York; 10 Department of Urology, University of Texas Southwestern, Dallas; 11 Department of Urology, Second Faculty of Medicine, Charles University, Prague, Czech Republic; 12 Hourani Center for Applied Scientific Research, Al-Ahliyya Amman University, Amman, Jordan; 13 Karl Landsteiner Institute of Urology and Andrology, Vienna, Austria

## Abstract

**Importance:**

Circadian rhythms affect immunity, which could affect the effectiveness of immune checkpoint inhibitor (ICI). Whether the time of ICI administration is associated with clinical outcomes in advanced cancers remains unclear.

**Objective:**

To evaluate the association between time of day of ICI administration and oncologic outcomes in patients with advanced solid tumors.

**Data Sources:**

MEDLINE (via PubMed), Embase, and Web of Science Core Collection were searched in February 2026 to identify eligible studies.

**Study Selection:**

Randomized clinical trials and prospective or retrospective cohort studies were included that compared early vs late time-of-day ICI administration and reported overall survival (OS) and progression-free survival (PFS).

**Data Extraction and Synthesis:**

This systematic review and meta-analysis adhered to the Preferred Reporting Items for Systematic Reviews and Meta-Analyses reporting guideline. Two authors independently extracted data and assessed risk of bias. Random effects meta-analyses with inverse-variance method were performed.

**Main Outcomes and Measures:**

The primary outcomes were OS and PFS. These outcomes were reported as hazard ratios (HRs) with 95% CIs for early vs late ICI administration.

**Results:**

Of the 7892 records screened, 29 studies with 6129 patients were included. Among these studies, 1 was a randomized clinical trial in non–small cell lung cancer (NSCLC; 210 patients), 1 was a prospective cohort study in head and neck squamous cell carcinoma (62 patients), and 27 were retrospective cohort studies (5857 patients) across NSCLC, melanoma, gastric cancer, head and neck squamous cell carcinoma, renal cell carcinoma (RCC), esophageal cancer, small cell lung cancer, urothelial carcinoma, biliary tract cancer, hepatocellular carcinoma, and other cancers. Earlier ICI administration was associated with increased OS (HR, 0.60; 95% CI, 0.51-0.70) and PFS (HR, 0.62; 95% CI, 0.54-0.71). Subset analyses by cancer type confirmed significantly increased OS and PFS in NSCLC (OS: HR, 0.58 [95% CI, 0.46-0.74]; PFS: HR, 0.60 [95% CI, 0.46-0.76]), gastric cancer (OS: HR, 0.61 [95% CI, 0.49-0.77]; PFS: HR, 0.62 [95% CI, 0.43-0.89]), RCC (OS: HR, 0.60 [95% CI, 0.40-0.90]; PFS: HR, 0.70 [95% CI, 0.50-0.98]), small cell lung cancer (OS: HR, 0.37 [95% CI, 0.26-0.53]; PFS: HR, 0.48 [95% CI, 0.36-0.65]), and biliary tract cancer (OS: HR, 0.62 [95% CI, 0.41-0.93]; PFS: HR, 0.55 [95% CI, 0.38-0.79]).

**Conclusions and Relevance:**

In this systematic review and meta-analysis of studies including patients with advanced cancers, early immunotherapy administration was associated with improved outcomes. These findings suggest that treatment timing may have clinical relevance and warrant prospective evaluation to establish standardized timing strategies across cancer settings.

## Key Points

**Question:**

Is the time of day of immunotherapy administration associated with clinical outcomes in patients with advanced cancers?

**Findings:**

In this systematic review and meta-analysis of 29 studies involving 6129 patients, early time-of-day immunotherapy was associated with increased overall survival and progression-free survival. This survival benefit was confirmed by a randomized clinical trial for non–small cell lung cancer, while evidence from retrospective cohort studies indicated an association between early administration and improvement in gastric, renal cell, small cell lung, and biliary tract cancers.

**Meaning:**

The findings of this study suggest that earlier time-of-day immunotherapy is associated with increased survival and warrant prospective evaluation for clinical implementation.

## Teaser

This systematic review and meta-analysis assesses overall survival, progression-free survival, and adverse events associated with early vs late timing of immunotherapy across different cancer settings.

## Introduction

Immune checkpoint inhibitors (ICIs) are drugs that block inhibitory checkpoint pathways, such as programmed cell death 1 (PD-1), programmed cell death ligand 1 (PD-L1), and cytotoxic T-lymphocyte antigen-4 (CTLA-4), to enhance antitumor T-cell activity.^1^ ICIs represent a paradigm shift in cancer treatment and are now standard of care for various cancers.^1,2^ Circadian rhythm, an intrinsic biological clock synchronized with the day-night cycle, regulates numerous physiological processes, including immune functions. The number and function of immune cells in the tumor microenvironment have been shown to fluctuate with circadian rhythms,^3,4^ similar to the expression of immune checkpoint molecules, including PD-L1 and CTLA-4, suggesting a possible role in tumor immune evasion.^3,5,6^

These findings led to the concept of chronotherapy, which aims to improve treatment effectiveness by aligning drug administration timing with circadian rhythms.^7,8^ Several studies, including a systematic review,^9^ have explored the associations between the time of day of immunotherapy and survival outcomes across different malignant neoplasms. Most of the available evidence has come from retrospective studies, and only 1 randomized clinical trial (RCT) has been reported to date.^10^ However, previous reviews included fewer studies and did not comprehensively examine differences in cancer types, ICI regimens, concomitant therapies, and definitions of early vs late administration.

Therefore, we conducted this systematic review and meta-analysis to evaluate the association between time of day of ICI administration and oncologic outcomes in patients with advanced solid tumors. Specifically, we sought to clarify the clinical utility of chronomodulated immunotherapy.

## Methods

The study protocol was registered in PROSPERO (CRD420251076548). We followed the Preferred Reporting Items for Systematic Reviews and Meta-Analyses (PRISMA) reporting guideline.^11^

### Search Strategy

In February 2026, a comprehensive literature search of MEDLINE (via PubMed), Embase, and Web of Science Core Collection was conducted for studies investigating the association between the time of day of ICI administration and oncological outcomes in patients with advanced cancer. The detailed search strategy appears in eMethods 1 in Supplement 1. References were merged, deduplicated, and prepared for screening using reference and citation management software (EndNote 21.5; Clarivate). Two investigators (S.I. and K.M.) independently screened titles and abstracts. Relevant studies underwent full-text review, and reasons for exclusion were noted. We also manually searched the reference lists for additional studies. Disagreements were resolved by consensus among the coauthors.

### Inclusion and Exclusion Criteria

We defined the eligibility criteria using the PICOS (Population, Intervention, Comparison, Outcomes, and Study design) framework (eMethods 2 in Supplement 1).^12^ Studies assessing patients with advanced solid tumors receiving ICI therapy were included, whereas studies assessing patients with hematologic malignant neoplasms were excluded. In the included studies, ICI administration during a predefined early time of day was compared with ICI administration during a predefined late time of day.

The primary outcomes were overall survival (OS) and progression-free survival (PFS). Secondary outcomes included the incidence and profile of adverse events (AEs). Eligible study designs included RCTs and prospective or retrospective cohort studies. We excluded studies without original patient data as well as reviews, letters, editorial comments, author responses, case reports, case-control studies, and articles not published in English. For duplicate cohorts, we included the most recent eligible publication.

### Data Extraction

Two authors (S.I. and I.T.) independently extracted study and patient data, including the first author’s name, publication year, study design, country, recruitment period, cancer type, number of patients, patient age and sex, follow-up duration, performance status, ICI regimen, concomitant therapy, definition of early vs late administration, survival outcomes with hazard ratios (HRs) and 95% CIs, adjusted covariates in multivariable analyses, and AE outcomes. When relevant outcome measures were not directly reported, we used WebPlotDigitizer, version 5.2 (Automeris LLC) to digitize Kaplan-Meier curves and extract survival data.^13^ Disagreements during data extraction were resolved through discussion among coauthors.

### Risk-of-Bias Assessment

Risk of bias (ROB) was assessed using the ROBINS-I tool, version 2, for nonrandomized studies^14^ and ROB 2 tool^15^ for RCTs (Cochrane). The assessments for each study were independently conducted by 2 of us (S.I. and K.M.).

### Statistical Analysis

Studies reporting survival outcomes without HRs were included in qualitative synthesis but not in quantitative meta-analysis. Quantitative syntheses were performed using the meta package in R, version 4.5.0 (R Project for Statistical Computing). We used random effects models with DerSimonian-Laird inverse-variance method. We pooled log-HRs with corresponding SEs derived from 95% CIs. When multiple models were reported, the most fully adjusted HR was selected. If adjusted estimates were not available, unadjusted HRs were used.

The results were presented on forest plots with back-transformed HRs and corresponding 95% CIs. Heterogeneity was assessed using Cochran Q test and the *I*^2^ statistic (≥50% considered significant). When significant heterogeneity was observed, we performed sensitivity analyses. Sensitivity analyses consisted of a leave-one-out procedure applied to the whole dataset, including studies at high ROB. Publication bias was assessed using funnel plots. The Egger test was performed when 10 or more studies were included in each analysis. Exploratory subgroup analyses were conducted by definitions of early vs late time-of-day administration (clock-time cutoff and infusion proportion) and by ICI regimen type. Two-sided *P* < .05 were considered statistically significant. All tests were 2-sided.

## Results

### Study Selection and Characteristics

**Figure 1.**
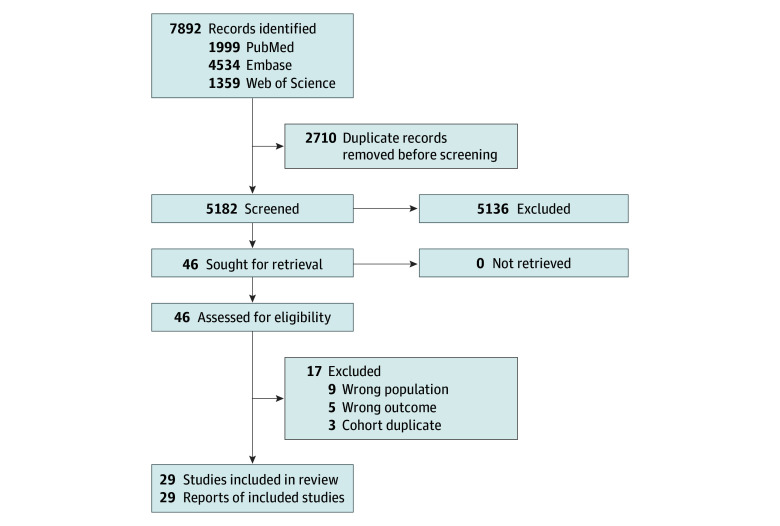
Flowchart of the Article Selection Process

The PRISMA flow diagram is provided in Figure 1. From 7892 screened individual records, we identified 29 studies comprising 6129 patients.^10,16,17,18,19,20,21,22,23,24,25,26,27,28,29,30,31,32,33,34,35,36,37,38,39,40,41,42,43^ Study types were 1 RCT (210 patients),^10^ 1 prospective cohort study (62 patients),^33^ and 27 retrospective cohort studies (5857 patients).^16,17,18,19,20,21,22,23,24,25,26,27,28,29,30,31,32,34,35,36,37,38,39,40,41,42,43^ Characteristics of and oncological outcomes from the included studies are summarized in Table 1 and Table 2.

**Table 1.** Basic Characteristics of Included Studies

Source	Design; country	Cancer type	No. of patients	Follow-up duration, mo	No. (%)		Definition of early vs late time-of-day ICI administration	Method for determining early vs late cutoff	Outcomes available
					ICI agents^a^	Concomitant therapy			
Nepote et al,^16^ 2025	Single-center, retrospective; Switzerland	Stage III or IV melanoma	Total:41; early: 21; late: 20	Median (range): 29.7 (3.1-60.4)	Ipilimumab + nivolumab: 41 (100)	None	Early: ≥50% of infusions before 14:00; late: <50% of infusion before 14:00	NA	OS, PFS, AE
Huang Z et al,^10^ 2026	RCT (LungTIME-C01 trial); China	Stage IIIC-IV NSCLC	Total: 210; early: 105; late: 105	Median (range): 28.7 (1.4-35.2)	Pembrolizumab: 48 (23); sintilimab: 162 (77)	Chemotherapy (carboplatin + nab-paclitaxel/carboplatin + pemetrexed): 210 (100)	Early: Infusion before 15:00; late: Infusion after 15:00	Time point yielding the lowest HR for PFS among candidate cutoff values	OS, PFS, AE
Tsukaguchi et al,^17^ 2025	Multicenter, retrospective; Japan	Unresectable NSCLC	Total: 290; early: 115; late: 175	Median (95% CI): 62.5 (56.4-67.9)	Pembrolizumab: 290 (100)	None	Early: first infusion before 11:00; late: first infusion after 11:00	Based on a preclinical study suggesting ICI effectiveness during early active phase (Tsuruta et al^44^)	OS, PFS, AE
Naganuma et al,^18^ 2025	Multicenter, retrospective; Japan	Unresectable hepatocellular carcinoma	Total: 751; early: 351; late: 400	NA	Atezolizumab: 751 (100)	Anti-VEGF inhibitor (bevacizumab): 751 (100)	Early: ≥80% of infusions before 12:00; late: ≥80% of infusions after 12:00	NA	OS, PFS
McMillan et al,^19^ 2025	Single-center, retrospective; US	Unresectable stage III NSCLC	Total: 178; early: 155; late: 23	Median: 48	Durvalumab: 178 (100)	Radiotherapy: 178 (100)	Early: ≤50% of infusions within 3 h of sunset; late: >50% of infusions within 3 h of sunset	NA	OS, PFS
Iwahashi et al,^20^ 2025	Multicenter, retrospective; Japan	Metastatic NSCLC	Total: 257; early: 51; late: 206	Median (95% CI): 75.9 (66-92.6)	Nivolumab: 257 (100)	None	Early: ≥2 of the first 3 infusions before 11:00; late: ≥2 of the first 3 infusions after 11:00	NA	OS, PFS
Ishizuka et al,^21^ 2025	Single-center, retrospective; Japan	Advanced unresectable or recurrent gastric cancer	Total: 248; early: 140; late: 108	Median (IQR): 9 (4.5-15.3)	Nivolumab: 248 (100)	None	Early: ≥70% of infusions before 14:00; late: <70% of infusions before 14:00	Time interval with the highest No. of infusions (13:30-14:00)	OS, PFS, AE
Huang Z et al,^22^ 2025 (December)	Single-center, retrospective; China	Extensive-stage small cell lung cancer	Total: 397; early: 344; late: 53	Median (range): 59 (35-78)	Atezolizumab: 223 (56); durvalumab: 174 (44)	Chemotherapy (platinum + etoposide/carboplatin + paclitaxel): 397 (100)	Early: ≥50% of infusions before 15:00; late: ≥50% of infusions after 15:00	Candidate cutoff times (11:00-16:00) evaluated using Cox proportional hazards regression models; cutoff with the lowest HR selected	OS, PFS
Huang Z et al,^23^ 2025 (March)	Single-center, retrospective; France and China	Locally advanced or metastatic NSCLC	Total: 713; early: 345; late: 368	Median (95% CI): 24.5 (23.2-25.6)	Pembrolizumab: 360 (51); sintilimab: 202 (28); tirelizumab: 55 (8); camrelizumab: 53 (7); toripalimab: 43 (6)	Chemotherapy (platinum + pemetrexed/carboplatin + paclitaxel): 713 (100)	Early: infusion before 11:30; late: infusion after 11:30	Modeled as continuous variable; administration timing showed decreasing risk in morning and increasing risk in afternoon, with inflection point at 11:00-12:00	OS, PFS
Gonçalves et al,^24^ 2025	Multicenter, retrospective; Portugal	Advanced urothelial carcinoma	Total: 105; early: 94; late: 11	Median (range): 17.7 (0.5-41.6)	Avelumab: 105 (100)	None	Early: <75% of infusions after 14:00; late: ≥75% of infusions after 14:00	Based on previous study by Gonçalves et al^40^	OS, PFS, AE
Gomez-Randulfe et al,^25^ 2025	Single-center, retrospective; UK	Advanced NSCLC	Total: 349; early: 188; late: 161	Median (IQR): 27.3 (13.1-46.4)	Pembrolizumab: 288 (83); atezolizumab: 61 (17)	None	Early: first infusion before 14:30; late: first infusion after 14:30	Aligned with the standard infusion schedule at the institution	OS, PFS, AE
Ersoy,^26^ 2025	Single-center, retrospective; Türkiye	Stage IV NSCLC	Total: 54; early: 30; late: 24	NA	Nivolumab: 54 (100)	None	Early: infusion before 13:00; late: infusion after 13:00	Based on studies by Karaboué et al^42^ and Tanaka et al^30^	PFS, AE
Cheng et al,^27^ 2025	Single-center, retrospective; China	Locally advanced or metastatic gastric cancer	Total: 214; early: 164; late: 50	Median: early, 14.6; late, 15.1	Sintilimab: 160 (75); nivolumab: 28 (13); tislelizumab, pembrolizumab, toripalimab, or camrelizumab: 26 (12)	FOLFOX-SOX-CAPOX chemotherapy: 214 (100)	Early: <20% of infusions after 16:30; late: ≥20% of infusions after 16:30	Based on MEMOIR study by Qian et al^43^	OS, PFS, AE
Amici et al,^28^ 2025	Single-center, retrospective; France	Unresectable stage III or IV melanoma	Total: 154; early: 56; late: 98	NA	Ipilimumab + nivolumab: 35 (23); ipilimumab: 10 (6); nivolumab or pembrolizumab: 109 (71)	None	Early: ≥50% infusions before 11:00; late: <50% infusions before 11:00	Based on median administration time across treatment courses	OS, PFS, AE
Zheng et al,^29^ 2024	Single-center, retrospective; China	Unresectable or advanced biliary tract cancer	Total: 221; early: 170; late: 51	Median: early, 10.5; late, 8.3	Durvalumab: 59 (27); sintilimab: 50 (23); camrelizumab: 25 (11); pembrolizumab: 20 (9); toripalimab: 20 (9); other agents: 47 (21)	Chemotherapy: 192 (87)	Early: <20% of infusions after 16:30; late: ≥20% of infusions after 16:30	Based on MEMOIR study by Qian et al^43^	OS, PFS, AE
Tanaka et al,^30^ 2024	Single-center, retrospective; Japan	Stage IV gastric cancer	Total: 58; early: 29; late: 29	NA	Nivolumab: 58 (100)	None	Early: median start time of infusion before 11:41; late: median start time of infusion after 11:41	Median value of all cases	OS, PFS, AE
Ruiz-Torres et al,^31^ 2024	Single-center, retrospective; US	Recurrent, advanced or metastatic HNSCC	Total: 98; early: 49; late: 49	Median (IQR): 13.2 (9.6-20.4)	Pembrolizumab: 81 (83); nivolumab: 11 (11); ipilimumab + nivolumab: 5 (5); durvalumab: 1 (1)	Chemotherapy: 13 (13)	Early: <20% of infusions after 15:00; late: ≥20% of infusions after 15:00	Based on previously defined cutoffs in chronomodulation or chronotherapy studies	OS, PFS, AE
Patel et al,^32^ 2024	Multicenter, retrospective; US	Metastatic RCC	Total: 201; early: 101; late: 100	Median (IQR): 18 (5-30)	Nivolumab: 117 (58); ipilimumab + nivolumab: 69 (34); pembrolizumab: 15 (8)	None	Early: ≥20% of infusions before 12:00; late: <20% of infusions before 12:00	Nearest h to the median infusion time among patients	OS, PFS
Janopaul-Naylor et al,^33^ 2024	Single-center, prospective, phase 2; US	Metastatic HNSCC	Total: 62; early: 34; late: 28	NA	Nivolumab: 62 (100)	Radiotherapy: 32 (52)	Early: <20% of infusions after 16:30; late: ≥20% of infusions after 16:30	Based on MEMOIR study by Qian et al^43^	OS, PFS, AE
Huang S et al,^34^ 2025	Multicenter, retrospective; China	Locally advanced esophageal cancer	Total: 120; early: 60; late: 60	NA	Camrelizumab: 44 (37); pembrolizumab: 29 (24); tislelizumab: 18 (15); sintilimab: 27 (22); other agents: 2 (2)	Chemotherapy (paclitaxel + platinum-based drugs): 120 (100)	Early: <75% of infusions after 12:00; late: ≥75% of infusions after 12:00	NA	OS
Hirata et al,^35^ 2024	Single-center, retrospective; Japan	Locally recurrent unresectable or locally advanced NSCLC	Total: 82; early: 70; late: 12	Median (IQR): 21 (12-36)	Durvalumab: 82 (100)	None	Early: <20% of infusions after 15:00; late: ≥20% of infusions after 15:00	NA	OS, PFS, AE
Catozzi et al,^36^ 2024	Single-center, retrospective; France	Overall	Total: 361; early: 136; late: 225	Median (95% CI): 32.2 (12.0-42.6)	Pembrolizumab: 186 (51); nivolumab: 132 (37); durvalumab: 22 (6); atezolizumab: 20 (6); ipilimumab: 1 (0)	Chemotherapy: 67 (19)	Early: infusion before 11:37; late: infusion after 11:37	Optimal cutoff for OS estimated with predictiveness curve method	OS, AE
Catozzi et al,^36^ 2024	Single-center, retrospective; France	Unresectable locally advanced or metastatic NSCLC	Total: 289; early: 113; late: 176	NA	NA	NA	Early: infusion before 11:37; late: infusion after 11:37	Optimal cutoff for OS estimated with predictiveness curve method	OS, AE
Catozzi et al,^36^ 2024	Single-center, retrospective; France	Other metastatic cancers (colorectal, melanoma, ENT, breast, urinary, or pancreatic)	Total: 72; early: 23; late: 49	NA	NA	NA	Early: infusion before 11:37; late: infusion after 11:37	Optimal cutoff for OS estimated with predictiveness curve method	OS, AE
Yeung et al,^37^ 2023	Single-center, retrospective; Canada	Unresectable or metastatic melanoma	Total: 121; early: 98; late: 23	Median (range): 15.4 (0.2-74.5)	Single ICI: 91 (75); dual ICIs: 30 (25)	None	Early: ≥1 of first 4 infusions before 13:00; late: All first 4 infusions after 13:00	NA	OS, PFS, AE
Rousseau et al,^38^ 2023	Single-center, retrospective; France	Advanced NSCLC	Total: 180; early: 115; late: 65	Median (95% CI): 42.9 (37.5-49.4)	Nivolumab, pembrolizumab, atezolizumab	None	Early: <20% of infusions after 16:30; late: ≥20% of infusions after 16:30	Based on MEMOIR study by Qian et al^43^	OS, PFS
Nomura et al,^39^ 2023	Single-center, retrospective; Japan	Recurrent or metastatic esophageal cancer	Total: 62; early: 27; late: 35	Median (range): 13.8 (2.5-60.2)	Nivolumab: 62 (100)	None	Early: first infusion before 13:00; late: first infusion after 13:00	Midpoint of infusion start times (10:00-16:00)	OS, PFS, AE
Gonçalves et al,^40^ 2023	Single-center, retrospective; Portugal	Stage IV melanoma	Total: 73; early: 48; late: 25	Median: 15.3	Nivolumab, pembrolizumab, ipilimumab + nivolumab	None	Early: <75% of infusions after 14:00; late: ≥75% of infusions after 14:00	NA	OS, PFS, AE
Dizman et al,^41^ 2023	Multicenter, retrospective; US	Metastatic RCC	Total: 135; early: 89; late: 46	Median (95% CI): 22.5 (18.4-25.8)	Ipilimumab + nivolumab: 69 (51); nivolumab: 66 (49)	None	Early: <20% of infusions after 16:30; late: ≥20% of infusions after 16:30	NA	OS
Karaboué et al,^42^ 2022	Single-center, retrospective; France	Locally advanced or metastatic NSCLC	Total: 95 early: 48; late: 47	NA	Nivolumab: 95 (100)	None	Early: ≥50% of infusions before 12:55; late: ≥50% of infusions after 12:55	Median clock-time of all treatment courses	OS, PFS, AE
Qian et al,^43^ 2021	Single-center, longitudinal, retrospective analysis (MEMOIR study); US	Stage IV melanoma	Total: 299; early: 225; late: 74	Median (IQR): 27 (14-47)	Pembrolizumab, ipilimumab, nivolumab, ipilimumab + nivolumab	None	Early: <20% of infusions after 16:30; late: ≥20% of infusions after 16:30	Mean of each infusion period	OS, PFS, AE

Abbreviations: AE, adverse event; CAPOX, capecitabine and oxaliplatin; ENT, ear-nose-throat; FOLFOX, folinic acid, fluorouracil, and oxaliplatin; HNSCC, head and neck squamous cell carcinoma; HR, hazard ratio; ICI, immune checkpoint inhibitor; NA, not available; NSCLC, non–small cell lung cancer; OS, overall survival; PFS, progression-free survival; RCC, renal cell carcinoma; RCT, randomized clinical trial; SOX, S-1 and oxaliplatin; VEGF, vascular endothelial growth factor.

^a^ The number of patients treated with each ICI agent was not reported in Rousseau et al,^38^ Gonçalves et al,^40^ and Qian et al.^43^

**Table 2.** Clinical Characteristics and Oncological Outcomes of Patients in Included Studies

Source	Age, y	Patients, No. (%)		Survival outcomes		AE outcomes among early and late ICI administration groups
		Sex	ECOG PS score	Outcome measure, HR (95% CI)^a^	Adjusted covariates	
Nepote et al,^16^ 2025	NA	Male: 23 (56); female: 18 (44)	0-1: 37 (90); ≥2: 4 (10)	OS: 0.22 (0.05-0.88); PFS: 0.35 (0.26-0.5)	No. of metastatic sites, *BRAF* V600 carrier status	Similar overall irAE incidence between the 2 groups; grade ≥2 irAEs requiring immunosuppressive treatment more common in the late vs early group (80% vs 52%)
Huang Z et al,^10^ 2026	NA	Male: 190 (90); female: 20 (10)	0: 70 (33); 1: 140 (67)	OS: 0.4 (0.28-0.58); PFS: 0.36 (0.26-0.5)	Age, sex, ECOG PS score, histological type, brain metastasis, liver metastasis, PD-L1 TPS, ICI agent, LIPI score	No AEs leading to death or treatment discontinuation in either group; no significant differences in irAE incidence between the 2 groups
Tsukaguchi et al,^17^ 2025	Median (IQR): 75 (68-80)	Male: 222 (77); female: 68 (23)	0-1: 243 (84); ≥2: 47 (16)	OS: 0.67 (0.46-0.97); PFS: 0.8 (0.59-1.1)	Age, sex, BMI, smoking status, ECOG PS score, disease stage, CNS metastasis, liver metastasis, histological type, PD-L1 TPS, corticosteroid use, antibiotics use, PPI use, NLR	Grade ≥3 irAEs more frequent in the early vs late group (27 [26.2%] vs 14 [13.6%])
Naganuma et al,^18^ 2025	Median (range): 74 (68-79)	Male: 596 (79); female: 155 (21)	0: 611 (81); 1: 115 (15); 2: 25 (3)	Median OS, mo: early, 24.7; late, 21.4; PFS: 0.81 (0.69-0.96)	Age, sex, etiological factor, ECOG PS score, Child-Pugh score, portal vein tumor thrombosis, extra hepatic metastasis, AFP, DCP, NLR	NA
McMillan et al,^19^ 2025	Median (IQR): 67.5 (61-74)	Male: 105 (59); female: 73 (41)	0: 90 (51); 1: 88 (49)	OS: 0.62 (0.3-1.25); PFS: 0.60 (0.32-1.11)	Age, ECOG PS score, TMB, RT dose	NA
Iwahashi et al,^20^ 2025	Median (IQR): 70 (64-75)	Male: 173 (67); female: 84 (33)	0: 51 (20); 1: 165 (64); ≥2: 41 (16)	OS: 0.75 (0.52-1.09); PFS: 0.77 (0.55-1.06)	OS: histological type, ECOG PS score, brain metastasis, liver metastasis, pleural dissemination, concomitant corticosteroid use, CRP, LDH, NLR; PFS: unadjusted	NA
Ishizuka et al,^21^ 2025	NA	Male: 159 (64); female: 89 (36)	0: 62 (25); ≥1: 186 (75)	OS: 0.67 (0.5-0.9); PFS: 0.70 (0.53-0.92)	Age, histological type, ECOG PS score, ascites, NLR, mGPS	Any irAE, early vs late: 57 (41%) vs 32 (30%); grade ≥3 irAEs, early vs late: 10 (7%) vs 6 (6%)
Huang Z et al,^22^ 2025 (December)	Median (range): 59 (35-78)	Male: 355 (89); female: 42 (11)	0: 89 (22); 1: 308 (78)	OS: 0.37 (0.27-0.53); PFS: 0.48 (0.36-0.65)	Sex, age, smoking history, ECOG PS score, liver metastasis, brain metastasis, ICI agent	NA
Huang Z et al,^23^ 2025 (March)	Median (IQR): 62 (56-68)	Male: 598 (84); female: 115 (16)	0: 130 (18); 1: 535 (75); 2: 46 (7); missing: 2 (0)	OS: 0.47 (0.37-0.60); PFS: 0.54 (0.45-0.65)	Ethnicity, sex, age, ICI agent, chemotherapy protocol, WHO PS score, tumor stage, PD-L1 expression, No. of metastatic sites	NA
Gonçalves et al,^24^ 2025	Median (IQR): 70 (64-75)	Male: 82 (78); female: 23 (22)	0: 58 (55); 1: 42 (40); 2: 5 (5)	OS: 0.35 (0.12-1.00); PFS: 0.43 (0.18-1.03)	Age, sex, ECOG PS score, tumor grade, presence of visceral metastases	Any irAE, early vs late: 36 (38%) vs 10 (91%); grade ≥3 irAEs, early vs late: 5 (5%) vs 2 (18%)
Gomez-Randulfe et al,^25^ 2025	Median (IQR): 27.3 (13.1-46.4)	Male: 181 (52); female: 168 (48)	0: 47 (13); 1: 269 (77); 2: 33 (10)	OS: 0.88 (0.7-1.1); PFS: 0.92 (0.74-1.15)	OS: unadjusted; PFS: unadjusted	179 Patients (51%) had any irAE; 47 (14%) had grade ≥3 irAEs; no significant differences irAE incidence between the 2 groups
Ersoy,^26^ 2025	NA	Male: 48 (89); female: 6 (11)	0: 11 (20); 1: 34 (63); 2: 9 (17)	Median PFS, mo: early, 3.5; late, 3.2	Unadjusted	Any irAE, early vs late: 4 (13%) vs 6 (25%)
Cheng et al,^27^ 2025	NA	Male: 144 (67); female: 70 (33)	0: 150 (70); ≥1: 64 (30)	OS: 0.63 (0.42-0.95); PFS: 0.75 (0.49-1.15)	Concurrent drugs, primary tumor location, histological differentiation, MMR status, pretreatment CA19-9 and CA72-4 levels	Any AE, early vs late: 149 (91%) vs 45 (90%); grade ≥3 AEs, early vs late: 49 (30%) vs 14 (28%); any irAE, early vs late: 61 (37%) vs 22 (44%); grade ≥3 irAEs, early vs late: 2 (1%) vs 0 (0%)
Amici et al,^28^ 2025	Median (IQR): 67 (56-75)	Male: 91 (59); female: 63 (41)	0: 112 (74); 1: 38 (25); ≥2: 2 (1)	OS: 1.06 (0.72-1.59); PFS: 1.01 (0.72-1.41)	BMI, Breslow thickness, LDH, tumor burden, liver metastasis, bone metastasis, interval between surgery and first ICI infusion	Any AE, early vs late: 6 (11%) vs 33 (34%); grade ≥3 AEs, early vs late: 6 (11%) vs 28 (29%)
Zheng et al,^29^ 2024	Mean (SD): 58 (10.3)	Male: 117 (53); female: 104 (47)	0: 165 (75); ≥1: 56 (25)	OS: 0.62 (0.41-0.93); PFS: 0.55 (0.38-0.79)	Site of origin, histological differentiation, ICI agent, line of ICI therapy, smoking status, pretreatment CA19-9, CEA, and CA125 levels	No significant differences in any AEs, grade 3/4 AEs, any irAEs, grade 3/4 irAEs, and AEs leading to discontinuation of medication
Tanaka et al,^30^ 2024	NA	Male: 48 (83); female: 10 (17)	0: 24 (41); 1: 28 (48); 2: 6 (10)	OS: 0.34 (0.17-0.69); PFS: 0.36 (0.20-0.67)	Age, sex, ECOG PS score, irAE status, NSAIDs use	Any irAE, early vs late: 5 (17%) vs 6 (21%)
Ruiz-Torres et al,^31^ 2024	Median (range): 65 (28-96)	Male: 69 (70); female: 29 (30)	≤1: 100 (88); 2: 13 (12)	OS: 0.71 (0.56-0.83); PFS: 0.63 (0.41-0.98)	Disease stage, prior surgery, prior RT, prior chemotherapy, HPV status	Grade ≥3 irAEs, early vs late: 5 (10%) vs 2 (4%)
Patel et al,^32^ 2024	Median (IQR): 63 (56-70)	Male: 146 (73); female: 55 (27)	0: 86 (43); 1: 79 (39); ≥2: 36 (18)	OS: 0.57 (0.33-0.98); PFS: 0.70 (0.50-0.98)	Age, sex, ECOG PS score, IMDC risk category, pretreatment LDH, histological type, presence of bone, brain, and liver metastases	NA
Janopaul-Naylor et al,^33^ 2024	NA	NA	NA	OS: 1.44 (0.75-2.76); PFS: 1.15 (0.67-1.98)	Unadjusted	Grade ≥3 AEs, early vs late: 5 (15%) vs 2 (7%)
Huang S et al,^34^ 2025	Median (IQR): 62 (57-68)	Male: 97 (81); female: 23 (19)	NA	OS: 2.63 (1.22-5.88)	Surgery, infusion interval of ICI	NA
Hirata et al,^35^ 2024	Median (IQR): 69 (62-74)	Male: 67 (82); female: 15 (18)	0: 38 (46); 1: 41 (50); ≥2: 3 (4)	OS: 0.78 (0.31-1.96); PFS: 0.39 (0.18-0.97)	Age, sex, ECOG PS score, smoking status, histological subtype, PD-L1 expression, LIPI group, disease stage, No. of durvalumab infusions	AEs leading to permanent discontinuation of ICI, early vs late: 9 (13%) vs 2 (17)
Catozzi et al,^36^ 2024	Mean (SD): 62.5 (10.7)	Male: 222 (62); female: 139 (38)	0-1: 298 (83); 2-3: 62 (17)	OS, overall: 0.64 (0.48-0.85); OS, NSCLC: 0.63 (0.45-0.88); OS, other cancers: 0.68 (0.36-1.28)	Unadjusted	Any AE, early vs late: 67 (49%) vs 76 (34%); grade ≥3 AEs, early vs late: 9 (7%) vs 14 (6%)
Yeung et al,^37^ 2023	NA	Male: 76 (63): female: 45 (37)	≤1: 97 (80); ≥2: 24 (20)	OS: 0.42 (0.23-0.76); PFS: 0.50 (0.29-0.85)	ECOG PS score, sex, baseline prednisone dose, ICI regimen, brain metastases, LDH	Any irAE, early vs late: 71 (72%) vs 10 (43%)
Rousseau et al,^38^ 2023	Median (IQR): 65 (57-70)	Male: 112 (62); female: 68 (38)	0: 49 (27); 1: 91 (51); 2: 34 (19); 3: 6 (3)	OS: 0.68 (0.45-1.01); PFS: 0.69 (0.49-0.99)	Age, *EGFR* sequence variation status, metastatic sites, immunotherapy line, ECOG PS score, baseline corticosteroid use, NLR	NA
Nomura et al,^39^ 2023	NA	Male: 50 (81); female: 12 (19)	0: 30 (48); 1: 29 (47); 2: 3 (5)	OS: 0.39 (0.21-0.71); PFS: 0.40 (0.22-0.71)	Age, ECOG PS score, recurrent status	The most common AEs were pruritus and hypothyroidism. No grade 4/5 AEs were observed.
Gonçalves et al,^40^ 2023	Median (range): 70 (29-91)	Male: 45 (62); female: 28 (38)	0: 48 (66); 1: 25 (34)	OS: 0.45 (0.23-0.86); median PFS, mo: early, 14.9; late; 6.6	Unadjusted	Any irAE, early vs late: 32 (67%) vs 16 (64%)
Dizman et al,^41^ 2023	Median (range): 64 (31-89)	Male: 94 (70); female: 41 (30)	NA	OS: 0.63 (0.34-1.18)	Age, sex, IMDC risk category, ICI regimen, histological type, line of treatment	NA
Karaboué et al,^42^ 2022	Median (range): 67 (41-83)	Male: 79 (83); female: 16 (17)	0: 35 (37); 1: 56 (59); 2:4 (4)	OS: 0.17 (0.08-0.37); PFS: 0.26 (0.12-0.58)	Age, sex, WHO PS score, histological type, PD-L1 expression, No. of involved sites, prior RT, primary tumor resection status, line of treatment	Grade ≥3 fatigue more frequent in late vs early group (15% vs 6%); grade ≥2 skin toxic effect more frequent in early vs late group (32% vs 13%); pulmonary sepsis more frequent in the early vs late group (18% vs 7%)
Qian et al,^43^ 2021	Median (IQR): 61 (51-72)	Male: 197 (66); female: 102 (34)	0-1: 273 (91); 2: 22 (7); 3: 4 (1)	OS: 0.46 (0.24-0.91); PFS at 1 y after ICI initiation, early vs late: 56% vs 40%	Age, ECOG PS score, LDH, corticosteroid use, history of RT	AEs leading to discontinuation of ICIs, early vs late: 60 (27%) vs 20 (27%)

Abbreviations: AE, adverse event; AFP, α-fetoprotein; BMI, body mass index; CNS, central nervous system; CRP, C-reactive protein; DCP, des-γ-carboxy prothrombin; ECOG PS, Eastern Cooperative Oncology Group Performance Status; HPV, human papillomavirus; HR, hazard ratio; ICI, immune checkpoint inhibitor; IMDC, International mRCC (metastatic renal cell carcinoma) Database Consortium; irAE, immune-related adverse event; LDH, lactate dehydrogenase; LIPI, Lung Immune Prognostic Index; mGPS, modified Glasgow Prognostic Score; MMR, mismatch repair; NA, not available; NLR, neutrophil-to-lymphocyte ratio; NSAID, nonsteroidal anti-inflammatory drug; OS, overall survival; PD-L1, programmed cell death 1 ligand 1; PFS, progression-free survival; PPI, proton pump inhibitor; RT, radiotherapy; TMB, tumor mutational burden; TPS, tumor proportion score; WHO PS, World Health Organization Performance Status.

^a^ Naganuma et al,^18^ Ersoy,^26^ Gonçalves et al,^40^ and Qian et al^43^ did not report HRs for OS or PFS and were therefore not included in the meta-analyses.

Among the 29 studies, 11 (38%) included 2697 patients with non–small cell lung cancer (NSCLC),^10,17,19,20,23,25,26,35,36,38,42^ 5 (17%) included 688 patients with melanoma,^16,28,37,40,43^ 3 (10%) included 520 patients with gastric cancer,^21,27,30^ 2 (7%) included 160 patients with head and neck squamous cell carcinoma (HNSCC),^31,33^ 2 (7%) included 336 patients with renal cell carcinoma (RCC),^32,41^ 2 (7%) included 182 patients with esophageal cancer,^34,39^ 1 (3%) included 397 patients with small cell lung cancer,^22^ 1 (3%) included 105 patients with urothelial carcinoma,^24^ 1 (3%) included 221 patients with biliary tract cancer,^29^ and 1 (3%) included 751 patients with hepatocellular carcinoma.^18^ Catozzi et al^36^ included 7 cancer types and reported outcomes for NSCLC (289 patients) and pooled non-NSCLC cohort (72 patients). Twenty-one studies (72%) used single-agent ICI regimen,^10,17,18,19,20,21,22,23,24,25,26,27,29,30,33,34,35,36,38,39,42^ 1 (3%) used dual ICI regimen (ipilimumab plus nivolumab),^16^ and 7 (24%) included mixed cohorts of both single and dual ICI regimens.^28,31,32,37,40,41,43^ Eleven studies (28%) involved concomitant therapies with ICI: 8 with chemotherapy,^10,22,23,27,29,31,34,36^ 2 with radiotherapy,^19,33^ and 1 with an anti–vascular endothelial growth factor inhibitor.^18^ For the meta-analyses, data were pooled regardless of concomitant therapy status. Four studies (14%) reported OS or PFS without HRs and were therefore included in the qualitative synthesis only.^18,26,40,43^

### Assessment of Study Quality and ROB

ROB assessment of the included studies is summarized in eFigures 1 and 2 in Supplement 1. Two nonrandomized studies (7%) had serious ROB, and 26 (90%) had moderate ROB. Among studies rated as having serious ROB, Janopaul-Naylor et al^33^ was determined to have administration timing based on institutional workflow and patient preferences, which could introduce selection bias and confounding by clinical factors. For example, patients who have better fitness or are employed may obtain earlier treatment slots, while patients with poorer performance status tend to be treated later. Secondary analyses lacked stratification or multivariable adjustment, and imbalances between the early and late groups could not be excluded. In Gonçalves et al,^40^ confounding was not addressed with multivariable models, and baseline similarity was estimated using χ^2^ tests in a small cohort (n = 73), leaving residual and unmeasured confounding. The RCT by Huang et al^10^ was rated as having low ROB.

### Definitions of Early vs Late Time-of-Day ICI Administration

Operational definitions of early vs late time-of-day administration in each study are presented in Table 1. These definitions were grouped into 2 categories. Ten studies (34%) applied a simple clock-time cutoff (eg, infusion before vs after 15:00).^10,17,20,23,25,26,30,36,37,39^ Nineteen (66%) applied proportion-based definitions, classifying patients by the proportion of infusions delivered before or after a specified time threshold (eg, >80% of infusions before vs after 12:00; more vs less than 50% of infusions before 14:00).^16,18,19,21,22,24,27,28,29,31,32,33,34,35,38,40,41,42,43^ We accepted study-specific definitions for the primary meta-analysis and performed an exploratory subgroup analysis comparing the clock-time cutoff with the infusion-proportion approach.

### Meta-Analysis of Survival Outcomes

#### Overall Survival

**Figure 2.**
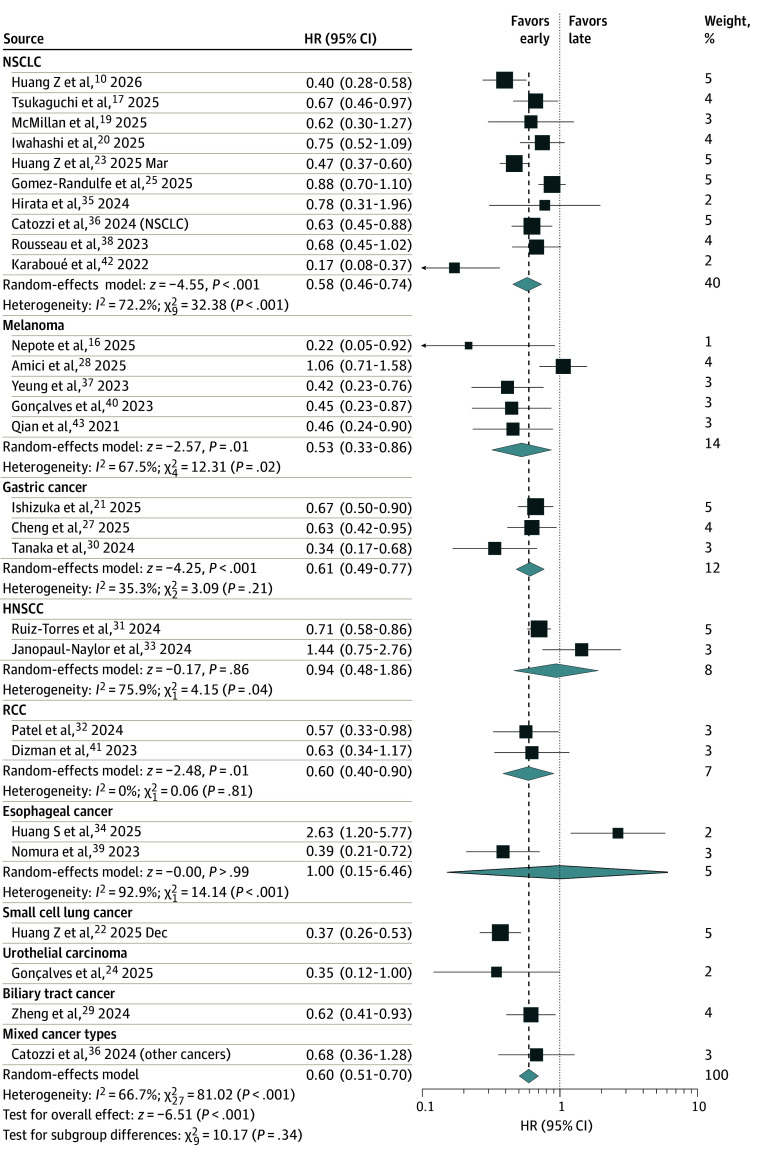
Forest Plot of the Association of Early vs Late Time-of-Day Administration of Immune Checkpoint Inhibitor With Overall Survival The size of the boxes represents the weight of each study in the meta-analysis. HNSCC indicates head and neck squamous cell carcinoma; HR, hazard ratio; NSCLC, non–small cell lung cancer; RCC, renal cell carcinoma.

Twenty-eight cohorts from 27 studies,^10,16,17,19,20,21,22,23,24,25,27,28,29,30,31,32,33,34,35,36,37,38,39,40,41,42,43^ including 5324 patients, reported HRs with 95% CIs for OS. Across these studies, early time-of-day ICI administration was associated with a significant increase in OS (HR, 0.60; 95% CI, 0.51-0.70; *P* < .001) (Figure 2). Stratified by cancer type, benefits from early administration were observed for NSCLC (n = 2643 patients; HR, 0.58; 95% CI, 0.46-0.74; *P* < .001), melanoma (n = 688 patients; HR, 0.53; 95% CI, 0.33-0.86; *P* = .01), gastric cancer (n = 520 patients; HR, 0.61; 95% CI, 0.49-0.77; *P* < .001), RCC (n = 336 patients; HR, 0.60; 95% CI, 0.40-0.90; *P* = .01), small cell lung cancer (n = 397 patients; HR, 0.37; 95% CI, 0.26-0.53; *P* = .001), and biliary tract cancer (n = 221 patients; HR, 0.62; 95% CI, 0.41-0.93; *P* = .02). The no association between early administration and increased OS was observed for urothelial carcinoma (n = 105 patients; HR, 0.35; 95% CI, 0.12-1.00; *P* = .05). No differences in OS were identified for HNSCC (n = 160 patients; HR, 0.94; 95% CI, 0.48-1.86; *P* = .86) and esophageal cancer (n = 182 patients; HR, 1.00; 95% CI, 0.15-6.46; *P* > .99).

Heterogeneity was statistically significant in the overall analysis (*I*^2^ = 67%; Q test *P* < .001). By cancer type, heterogeneity was substantial in NSCLC (*I*^2^ = 72%; Q test *P* < .001), melanoma (*I*^2^ = 68%; Q test *P* = .02), and HNSCC (*I*^2^ = 76%; Q test *P* = .04) but not in RCC. This heterogeneity could be attributable to differences in cancer type, ICI regimen, concomitant therapies, and definitions of early vs late time-of-day administration. Leave-one-out sensitivity analyses overall and of NSCLC studies did not identify a specific source of heterogeneity (eTable in Supplement 1). In melanoma, excluding the study by Amici et al^28^ produced a pooled HR of 0.42 (95% CI, 0.30-0.60) with no evidence of significant heterogeneity (*I*^2^ = 0%; Q test *P* = .83). Amici et al^28^ applied a markedly earlier cutoff time (11:00) than other melanoma cohorts, which could have played a role in the heterogeneity. In gastric cancer, excluding the study by Tanaka et al^30^ produced a pooled HR of 0.66 (95% CI, 0.52-0.83) with no evidence of significant heterogeneity (*I*^2^ = 0%; Q test *P* = .81). Tanaka et al^30^ applied an earlier cutoff time (11:41) than other cohorts and conducted the only study to define the cutoff using an infusion-proportion approach. This difference could have contributed to the heterogeneity. The funnel plot and Egger test showed no evidence of significant publication bias (eFigure 3 in Supplement 1). Sensitivity analysis limited to studies with adjusted HRs^10,16,17,19,20,21,22,23,24,27,28,29,30,31,32,34,35,37,38,39,41,42,43^ consistently showed increased OS associated with early time-of-day administration (HR, 0.57; 95% CI, 0.48-0.67) (eFigure 4 in Supplement 1). Analysis limited to studies with unadjusted HRs^25,33,36,40^ showed a similar direction of association, which did not reach statistical significance (HR, 0.76; 95% CI, 0.57-1.02) (eFigure 5 in Supplement 1).

#### Progression-Free Survival

**Figure 3.**
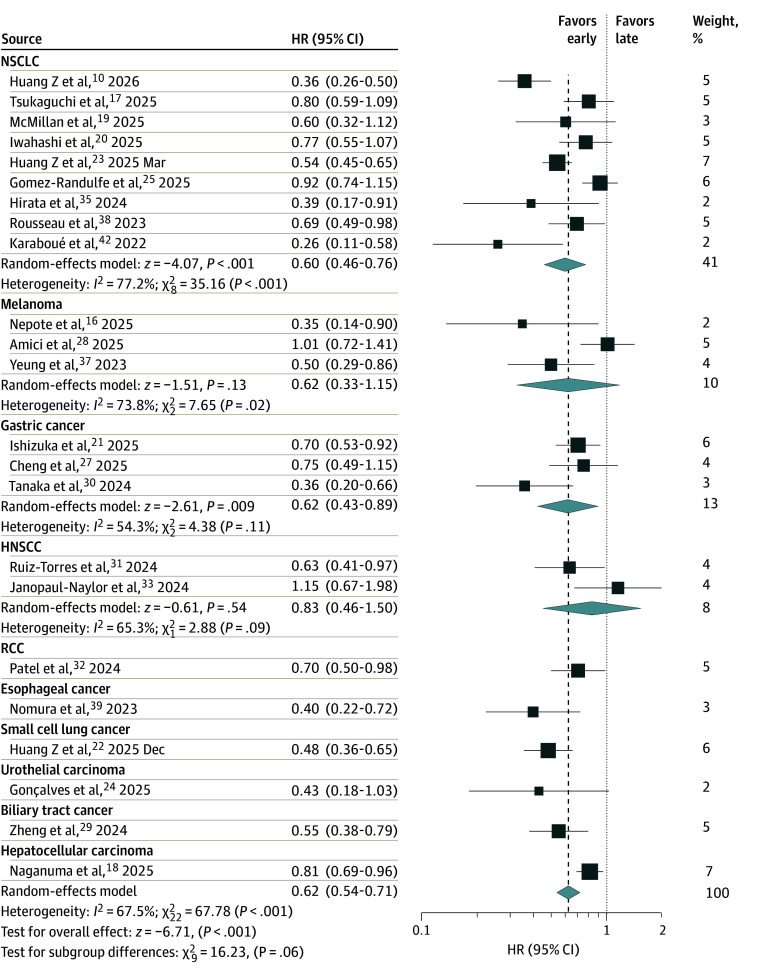
Forest Plot of the Association of Early vs Late Time-of-Day Administration of Immune Checkpoint Inhibitor With Progression-Free Survival The size of the boxes represents the weight of each study in the meta-analysis. HNSCC indicates head and neck squamous cell carcinoma; HR, hazard ratio; NSCLC, non–small cell lung cancer; RCC, renal cell carcinoma.

Twenty-three studies,^10,16,17,18,19,20,21,22,23,24,25,27,28,29,30,31,32,33,35,37,38,39,42^ including 5087 patients, reported HRs with 95% CIs for PFS. Pooled analysis showed a significant association of early administration with increased PFS (HR, 0.62; 95% CI, 0.54-0.71; *P* < .001) (Figure 3). By cancer type, NSCLC (n = 2354 patients; HR, 0.60; 95% CI, 0.46-0.76; *P* < .001), gastric cancer (n = 520 patients; HR, 0.62; 95% CI, 0.43-0.89; *P* = .01), RCC (n = 201 patients; HR, 0.70; 95% CI, 0.50-0.98; *P* = .04), esophageal cancer (n = 62 patients; HR, 0.40; 95% CI, 0.22-0.72; *P* = .002), small cell lung cancer (n = 397 patients; HR, 0.48; 95% CI, 0.36-0.65; *P* < .001), biliary tract cancer (n = 221 patients; HR, 0.55; 95% CI, 0.38-0.79; *P* = .001), and hepatocellular carcinoma (n = 751 patients; HR, 0.81; 95% CI, 0.69-0.96; *P* = .01) showed statistically significant benefit from early administration. The association did not reach conventional levels of statistical significance for urothelial carcinoma (n = 105 patients; HR, 0.43; 95% CI, 0.18-1.03; *P* = .05). No differences were identified for melanoma (n = 316 patients; HR, 0.62; 95% CI, 0.33-1.15; *P* = .13) and HNSCC (n = 160 patients; HR, 0.83; 95% CI, 0.46-1.50; *P* = .54).

There was statistically significant heterogeneity in the overall analysis (*I*^2^ = 68%; Q test *P* < .001). By cancer type, heterogeneity was substantial in NSCLC (*I*^2^ = 77%; Q test *P* < .001), melanoma (*I*^2^ = 74%; Q test *P* = .02), gastric cancer (*I*^2^ = 54%; Q test *P* = .11), and HNSCC (*I*^2^ = 65%; Q test *P* = .09). Heterogeneity was likely associated with the same factors as OS. Leave-one-out sensitivity analyses overall and of NSCLC studies did not identify a specific source (eTable in Supplement 1). For melanoma, heterogeneity disappeared when the study by Amici et al^28^ was excluded (*I*^2^ = 0%; Q test *P* = .52). For gastric cancer, heterogeneity disappeared when the study by Tanaka et al^30^ was excluded (*I*^2^ = 0%; Q test *P* = .79). The funnel plot and Egger test showed no evidence of significant publication bias (eFigure 3 in Supplement 1). Sensitivity analyses limited to studies with adjusted HRs (HR, 0.59; 95% CI, 0.50-0.69)^10,16,17,18,19,21,22,23,24,28,29,30,31,32,35,38,42^ (eFigure 6 in Supplement 1) and to studies with unadjusted HRs (HR, 0.74; 95% CI, 0.57-0.96)^20,25,27,33,37,39^ (eFigure 7 in Supplement 1) consistently showed increased PFS associated with early time-of-day administration.

### Exploratory Subgroup Analyses

#### Subgroup Analysis by Timing Definition Approach

In an analysis stratified by timing definitions of early vs late time-of-day administration, both clock-time cutoff and infusion proportion approaches consistently favored early administration for increased OS and PFS (eFigure 8 in Supplement 1). For OS, studies using simple clock-time cutoff (n = 2421 patients) produced a pooled HR of 0.56 (95% CI, 0.46-0.69; *P* < .001) with substantial heterogeneity (*I*^2^ = 67%; Q test *P* = .001).^10,17,20,23,25,30,36,37,39^ Studies using infusion proportion (n = 2903 patients) produced a pooled HR of 0.62 (95% CI, 0.49-0.79; *P* < .001) and similar heterogeneity as the other approach (*I*^2^ = 68%; Q test *P* < .001).^16,19,21,22,24,27,28,29,31,32,33,34,35,38,40,41,42,43^ There was no significant difference between the 2 definitions.

For PFS, studies using simple clock-time cutoff (n = 2060 patients) yielded a pooled HR of 0.57 (95% CI, 0.44-0.74; *P* < .001) with substantial heterogeneity (*I*^2^ = 80%; Q test *P* < .001),^10,17,20,23,25,30,37,39^ while studies with infusion proportion (n = 3027 patients) yielded a pooled HR of 0.66 (95% CI, 0.57-0.77; *P* < .001) with moderate heterogeneity (*I*^2^ = 53%; Q test *P* = .01).^16,18,19,21,22,24,27,28,29,31,32,33,35,38,42^ The difference between definitions was not significant.

#### Subgroup Analysis by ICI Regimen Type

Subgroup analyses according to ICI regimen type (single, dual, or mixed) showed that early time-of-day administration was associated with increased OS and PFS (eFigure 9 in Supplement 1). For OS, 19 studies using single ICI regimen (n = 4202 patients) produced a pooled HR of 0.60 (95% CI, 0.49-0.73; *P* < .001) with substantial heterogeneity (*I*^2^ = 72%; Q test *P* < .001).^10,17,19,20,21,22,23,24,25,27,29,30,33,34,35,36,38,39,42^ The study by Nepote et al^16^ reported greater OS associated with early time-of-day administration of dual ICI regimen (n = 41 patients; HR, 0.22; 95% CI, 0.05-0.92; *P* = .03). Seven studies, including both single and dual (mixed) regimens (n = 1081 patients), produced a pooled HR of 0.63 (95% CI, 0.49-0.81; *P* < .001).^28,31,32,37,40,41,43^ There were no significant differences among regimen types.

For PFS, 18 studies using single ICI regimen (n = 4472 patients) produced a pooled HR of 0.61 (95% CI, 0.52-0.71; *P* < .001) with substantial heterogeneity (*I*^2^ = 71%; Q test *P* < .001).^10,17,18,19,20,21,22,23,24,25,27,29,30,33,35,38,39,42^ The study by Nepote et al^16^ reported increased PFS with early time-of-day administration of dual ICI regimen (n = 41 patients; HR, 0.35; 95% CI, 0.14-0.90; *P* = .03). Four studies administering mixed regimens (n = 574 patients) produced a pooled HR of 0.72 (95% CI, 0.54-0.95; *P* = .02).^28,31,32,37^ There were no significant differences among regimen types.

### Adverse Events

Twenty studies reported AE outcomes.^10,16,17,21,24,25,26,27,28,29,30,31,33,35,36,37,39,40,42,43^ The details are summarized in Table 2. The pooled analysis was omitted due to inconsistent reporting of AEs. Overall, no consistent association was observed between time-of-day administration and incidence or severity of AEs.

## Discussion

In this systematic review and meta-analysis, early time-of-day ICI administration was associated with increased survival. Subgroup analyses demonstrated significant benefits in both OS and PFS for NSCLC, gastric cancer, RCC, small cell lung cancer, and biliary tract cancer. The association remained consistent in studies with both adjusted and unadjusted HRs and across multiple exploratory analyses, suggesting robustness of the findings. No clear association between administration timing and AEs emerged, with incidence inconsistently reported across studies. These findings suggest a potential clinical relevance of chronotherapy.

The biological mechanism likely reflects the interplay between circadian rhythms and the immune system. Immune cells contain intrinsic clocks that drive diurnal variation in functions such as cytokine production and phagocytosis.^45^ Patients with cancer sometimes show disrupted circadian biomarkers, such as rest-activity rhythms and cortisol patterns, which may be associated with poorer outcome through impaired immunity.^46,47^ Key antitumor cells follow diurnal patterns; effector CD8^+^ T-cells increase and peak from morning to early afternoon,^48^ and dendritic cells impose strict circadian control over antitumor responses, with diurnal dendritic cell migration affecting CD8^+^ T-cell responses and tumor growth.^49^ Within the tumor microenvironment, PD-1 expression on macrophages and cytotoxic T-cell infiltration also fluctuate according to circadian rhythm.^44^ Chronotherapy in chemotherapy showed comparable survival but reduced specific toxic effects compared with conventional chemotherapy,^50,51^ supporting the alignment of drug administration with circadian rhythms to spare normal tissues. Our findings are consistent with the hypothesis that chronobiological mechanisms may also be relevant in immunotherapy.

Previous studies have suggested a link between time of ICI administration and survival, but the evidence remained fragmented and lacked quantitative analysis.^45^ Our identification of a significant association in NSCLC aligns with a recent RCT that showed a survival advantage with early administration.^10^ In contrast, Huang et al^34^ reported an association between superior OS and late administration in esophageal cancer, hypothesizing that late administration precedes the nocturnal peak of circulating T-cells and B-cells, which may enhance overnight immunity. However, that retrospective study’s heterogeneous ICI agents, immunochemotherapy regimen (ICI plus paclitaxel and platinum), and infusion proportion approach complicate interpretation of the role of timing in survival.

Our meta-analysis suggests that optimizing ICI timing is a novel strategy to improve outcomes. However, clinical translation requires standardized timing definitions and consideration of patient-level chronobiological processes. In our exploratory analysis, both clock-time and infusion proportion definitions favored early ICI administration, with no significant difference between definitions. For practice, a simple clock-time approach is likely easier to implement. Individual circadian profiles may shift the optimal timing. Simple assessments, such as rest-activity questionnaires, could guide personalized scheduling. Future prospective trials should use standardized timing definitions and evaluate whether chronotype-guided scheduling is associated with improved survival.

### Limitations

This study has several limitations. First, most included studies were retrospective, introducing selection bias and unmeasured confounding such as differences in patient scheduling patterns, performance status, treatment line, or concomitant therapies. Second, primary OS and PFS analyses showed substantial heterogeneity that could not be fully explained by sensitivity analyses, suggesting that implications of timing may vary by patient characteristics, cancer type, or ICI agent. Third, the lack of a standardized definition of administration timing limits an evidence-based clinical cutoff. Finally, inconsistent and incomplete AE reporting prevented a meta-analysis of safety, leaving the role of timing in toxic effects unresolved. Future research should focus on multicenter RCTs with standardized timing definitions and comprehensive safety reporting to validate chronomodulated immunotherapy and enable safe clinical implementation.

## Conclusions

In this systematic review and meta-analysis of studies including patients with advanced cancers, we found that early time-of-day immunotherapy was associated with increased OS and PFS, particularly in NSCLC, RCC, gastric, small cell lung, and biliary tract cancer. Although evidence from RCTs is currently limited to NSCLC, our findings highlight the potential relevance of administration timing as a modifiable factor in cancer immunotherapy. Prospective studies are needed to determine whether the observed associations are causal and to define standardized timing strategies across different cancer settings.

## Notes

Supplement 1. **eMethods 1.** Search Strategy for Systematic Review and Meta-Analysis **eMethods 2.** Study Design According to the PICOS Framework **eTable.** Summary of Leave-One-Out Sensitivity Analysis **eFigure 1.** Risk of Bias Assessment According to ROBINS-I Tool **eFigure 2.** Risk of Bias Assessment According to RoB 2 Tool **eFigure 3.** Funnel Plots Assessing Publication Bias **eFigure 4.** Sensitivity Meta-Analysis Using Only Adjusted Hazard Ratios for Overall Survival **eFigure 5.** Sensitivity Meta-Analysis Using Only Unadjusted Hazard Ratios for Overall Survival **eFigure 6.** Sensitivity Meta-Analysis Using Only Adjusted Hazard Ratios for Progression-Free Survival **eFigure 7.** Sensitivity Meta-Analysis Using Only Unadjusted Hazard Ratios for Progression-Free Survival **eFigure 8.** Exploratory Subgroup Analysis by Definitions of Early Versus Late Time of Day **eFigure 9.** Exploratory Subgroup Analysis by ICI Regimen Type

Supplement 2.
Data Sharing Statement
