# Supplementary material for: Time-of-Day Immunotherapy Administration and Outcomes in Advanced Cancers: A Systematic Review and Meta-Analysis
Source: JAMA Netw Open. 2026 May 5;9(5):e2610815. doi: 10.1001/jamanetworkopen.2026.10815 (PMC13147196; doi:10.1001/jamanetworkopen.2026.10815)
Supplement: Supplement 1. — eMethods 1. Search Strategy for Systematic Review and Meta-Analysis eMethods 2. Study Design According to the PICOS Framework eTable. Summary of Leave-One-Out Sensitivity Analysis eFigure 1. Risk of Bias Assessment According to ROBINS-I Tool eFigure 2. Risk of Bias Assessment According to RoB 2 Tool eFigure 3. Funnel Plots Assessing Publication Bias eFigure 4. Sensitivity Meta-Analysis Using Only Adjusted Hazard Ratios for Overall Survival eFigure 5. Sensitivity Meta-Analysis Using Only Unadjusted Hazard Ratios for Overall Survival eFigure 6. Sensitivity Meta-Analysis Using Only Adjusted Hazard Ratios for Progression-Free Survival eFigure 7. Sensitivity Meta-Analysis Using Only Unadjusted Hazard Ratios for Progression-Free Survival eFigure 8. Exploratory Subgroup Analysis by Definitions of Early Versus Late Time of Day eFigure 9. Exploratory Subgroup Analysis by ICI Regimen Type [file jamanetwopen-e2610815-s001.pdf]

Supplemental Online Content

Inoue S, Tsuboi I, Miszczyk M, et al. Time-of-day immunotherapy administration and outcomes in advanced cancers: a systematic review and meta-analysis. *JAMA Netw Open*. 2026;9(5):e2610815. doi:10.1001/jamanetworkopen.2026.10815

eMethods 1. Search Strategy for Systematic Review and Meta-Analysis
eMethods 2. Study Design According to the PICOS Framework
eTable. Summary of Leave-One-Out Sensitivity Analysis
eFigure 1. Risk of Bias Assessment According to ROBINS-I Tool
eFigure 2. Risk of Bias Assessment According to RoB 2 Tool
eFigure 3. Funnel Plots Assessing Publication Bias
eFigure 4. Sensitivity Meta-Analysis Using Only Adjusted Hazard Ratios for Overall Survival
eFigure 5. Sensitivity Meta-Analysis Using Only Unadjusted Hazard Ratios for Overall Survival
eFigure 6. Sensitivity Meta-Analysis Using Only Adjusted Hazard Ratios for Progression-Free Survival
eFigure 7. Sensitivity Meta-Analysis Using Only Unadjusted Hazard Ratios for Progression-Free Survival
eFigure 8. Exploratory Subgroup Analysis by Definitions of Early Versus Late Time of Day
eFigure 9. Exploratory Subgroup Analysis by ICI Regimen Type

This supplemental material has been provided by the authors to give readers additional information about their work.

© 2026 Inoue S et al. *JAMA Network Open*

eMethods 1. Search strategy for systematic review and meta-analysis

Electronic searches were performed in the following databases to identify eligible studies.

1. MEDLINE (n = 1,999)
2. Embase (n = 4,534)
3. Web of Science (n = 1,359)

There were no language or publication period filters.

MEDLINE (via PubMed)

#	Search string	Results
#1	"Neoplasms"[MeSH Terms] OR "neoplasm*"[Title/Abstract] OR "cancer*"[Title/Abstract] OR "carcinoma*"[Title/Abstract] OR "tumor*"[Title/Abstract] OR "tumour*"[Title/Abstract] OR "malignan*"[Title/Abstract]	5,695,447
#2	"Immune Checkpoint Inhibitors"[MeSH Terms] OR "Immunotherapy"[MeSH Terms] OR "immunochemotherapy"[Title/Abstract] OR "immune checkpoint inhibitor*"[Title/Abstract] OR "pd 1 inhibitor*"[Title/Abstract] OR "pd l1 inhibitor*"[Title/Abstract] OR "ctla 4 inhibitor*"[Title/Abstract] OR "pembrolizumab"[Title/Abstract] OR "nivolumab"[Title/Abstract] OR "avelumab"[Title/Abstract] OR "ipilimumab"[Title/Abstract] OR "atezolizumab"[Title/Abstract] OR "durvalumab"[Title/Abstract] OR "cemiplimab"[Title/Abstract] OR "tislelizumab"[Title/Abstract] OR "tremelimumab"[Title/Abstract] OR "sintilimab"[Title/Abstract]	428,845
#3	"Chronotherapy"[MeSH Terms] OR "Circadian Rhythm"[MeSH Terms] OR "Time Factors"[MeSH Terms] OR "Drug Administration Schedule"[MeSH Terms] OR "chronotherap*"[Title/Abstract] OR "circadian"[Title/Abstract] OR "time-of-day"[Title/Abstract] OR "time of day"[Title/Abstract] OR "time of administration"[Title/Abstract] OR "timing of administration"[Title/Abstract] OR "administration time"[Title/Abstract] OR "administration timing"[Title/Abstract] OR "time of infusion"[Title/Abstract] OR "infusion time"[Title/Abstract] OR "infusion timing"[Title/Abstract] OR "early administration"[Title/Abstract] OR "earlier administration"[Title/Abstract] OR "late administration"[Title/Abstract] OR "later administration"[Title/Abstract] OR "early infusion"[Title/Abstract] OR "earlier infusion"[Title/Abstract] OR "late infusion"[Title/Abstract] OR "later infusion"[Title/Abstract] OR "morning"[Title/Abstract] OR "before noon"[Title/Abstract] OR "daytime"[Title/Abstract] OR "diurnal"[Title/Abstract] OR "afternoon"[Title/Abstract] OR "evening"[Title/Abstract]	1,563,889
#4	"Survival Rate"[MeSH Terms] OR "Survival Analysis"[MeSH Terms] OR "progression free survival"[MeSH Terms] OR "Treatment Outcome"[MeSH Terms] OR "overall survival"[Title/Abstract] OR "OS"[Title/Abstract] OR "progression-free survival"[Title/Abstract] OR "progression free survival"[Title/Abstract] OR "PFS"[Title/Abstract]	1,922,735
#5	#1 AND #2 AND #3 AND #4	1,999

Embase

#	Search string	Results
#1	'neoplasm'/exp OR cancer*:ti,ab OR carcinoma*:ti,ab OR tumor*:ti,ab OR tumour*:ti,ab OR malignan*:ti,ab	8,248,313
#2	'immune checkpoint inhibitor'/exp OR 'immunotherapy'/exp OR 'immunochemotherapy':ti,ab OR 'immune checkpoint inhibitor*':ti,ab OR 'anti pd 1':ti,ab OR 'anti pd1':ti,ab OR 'pd 1 inhibitor*':ti,ab OR 'pd1 inhibitor*':ti,ab OR 'pd l1 inhibitor*':ti,ab OR 'pdl1 inhibitor*':ti,ab OR 'ctla 4 inhibitor*':ti,ab OR 'ctla4 inhibitor*':ti,ab OR pembrolizumab:ti,ab OR nivolumab:ti,ab OR avelumab:ti,ab OR ipilimumab:ti,ab OR atezolizumab:ti,ab OR durvalumab:ti,ab OR cemiplimab:ti,ab OR tislelizumab:ti,ab OR tremelimumab:ti,ab OR sintilimab:ti,ab	529,143

© 2026 Inoue S et al. *JAMA Network Open*

#3	'chronotherapy'/exp OR 'circadian rhythm'/exp OR 'drug administration schedule'/exp OR 'chronotherap*':ti,ab OR 'circadian':ti,ab OR 'time-of-day':ti,ab OR 'time of day':ti,ab OR 'time of administration':ti,ab OR 'timing of administration':ti,ab OR 'administration time':ti,ab OR 'administration timing':ti,ab OR 'time of infusion':ti,ab OR 'infusion time':ti,ab OR 'infusion timing':ti,ab OR 'early administration':ti,ab OR 'earlier administration':ti,ab OR 'late administration':ti,ab OR 'later administration':ti,ab OR 'early infusion':ti,ab OR 'earlier infusion':ti,ab OR 'late infusion':ti,ab OR 'later infusion':ti,ab OR 'morning':ti,ab OR 'before noon':ti,ab OR 'daytime':ti,ab OR 'diurnal':ti,ab OR 'afternoon':ti,ab OR 'evening':ti,ab	1,866,266
#4	'overall survival'/exp OR 'progression free survival'/exp OR 'overall survival':ti,ab OR 'progression-free survival':ti,ab OR 'progression free survival':ti,ab	771,742
#5	#1 AND #2 AND #3 AND #4	4,534

Web of Science

#	Search string	Results
#1	TS=(neoplasm* OR cancer* OR carcinoma* OR tumor* OR tumour* OR malignan*)	5,697,611
#2	TS=("immune checkpoint inhibitor*" OR immunotherapy OR immunochemotherapy OR "PD 1 inhibitor*" OR "PD L1 inhibitor*" OR "CTLA 4 inhibitor*" OR pembrolizumab OR nivolumab OR avelumab OR ipilimumab OR atezolizumab OR durvalumab OR cemiplimab OR tislelizumab OR tremelimumab OR sintilimab)	298,100
#3	TS=(chronotherap* OR "circadian rhythm" OR "time factor*" OR "drug administration schedule*" OR circadian OR "time-of-day" OR "time of day" OR "time of administration" OR "timing of administration" OR "administration time" OR "administration timing" OR "time of infusion" OR "infusion time" OR "infusion timing" OR "early administration" OR "earlier administration" OR "late administration" OR "later administration" OR "early infusion" OR "earlier infusion" OR "late infusion" OR "later infusion" OR morning OR "before noon" OR daytime OR diurnal OR afternoon OR evening)	2,878,226
#4	TS=("survival rate" OR "survival analysis" OR "progression-free survival" OR "treatment outcome" OR "overall survival" OR OS OR PFS)	617,861
#5	#1 AND #2 AND #3 AND #4	1,359

eMethods 2. Study design according to the PICOS framework

**Population**[a,b]	Patients with advanced solid tumors receiving ICI therapy
**Intervention**[b]	Administration of ICI during pre-defined early time of day
**Comparison**[b]	Administration of ICI during pre-defined late time of day
**Outcome**[c]	Overall survival and progression-free survival (primary outcome)<br>Incidence and profile of adverse events (secondary outcome)
**Study design**	Randomized controlled trials, prospective and retrospective cohort studies

Abbreviations: PICOS, Population, Intervention, Comparison, Outcome, and Study design; ICI, immune checkpoint inhibitor

[a] Studies assessing patients with hematologic malignancies were excluded.

[b] ICIs of interest included PD-1, PD-L1, and CTLA-4 inhibitors.

[c] Progression-free survival was defined as the duration from first ICI administration to disease progression, death, or last follow-up without progression.

© 2026 Inoue S et al. *JAMA Network Open*

eTable. Summary of leave-one-out sensitivity analysis

(A) Overall survival

Subgroup	Excluded study	HR [95% CI]	Heterogeneity
Overall	Nepote et al. 2026	0.60 [0.52-0.71]	I^2 = 67%, Q = 78.98 (P < .001)
	Huang Z et al. 2026	0.61 [0.52-0.71]	I^2 = 65%, Q = 74.97 (P < .001)
	Tsukaguchi et al. 2025	0.59 [0.50-0.70]	I^2 = 68%, Q = 80.88 (P < .001)
	McMillan et al. 2025	0.60 [0.51-0.70]	I^2 = 68%, Q = 81.02 (P < .001)
	Iwahashi et al. 2025	0.59 [0.50-0.70]	I^2 = 68%, Q = 80.05 (P < .001)
	Ishizuka et al. 2025	0.59 [0.50-0.70]	I^2 = 68%, Q = 80.79 (P < .001)
	Huang Z et al. 2025 Dec	0.61 [0.53-0.71]	I^2 = 64%, Q = 71.84 (P < .001)
	Huang Z et al. 2025 Mar	0.61 [0.52-0.71]	I^2 = 65%, Q = 75.07 (P < .001)
	Gonçalves et al. 2025	0.60 [0.52-0.71]	I^2 = 67%, Q = 79.83 (P < .001)
	Gomez-Randulfe et al. 2025	0.59 [0.50-0.69]	I^2 = 63%, Q = 71.07 (P < .001)
	Cheng et al. 2025	0.60 [0.51-0.70]	I^2 = 68%, Q = 81.02 (P < .001)
	Amici et al. 2025	0.58 [0.50-0.68]	I^2 = 65%, Q = 73.93 (P < .001)
	Zheng et al. 2024	0.60 [0.51-0.70]	I^2 = 68%, Q = 81.02 (P < .001)
	Tanaka et al. 2024	0.61 [0.52-0.71]	I^2 = 67%, Q = 78.08 (P < .001)
	Ruiz-Torres et al. 2024	0.59 [0.50-0.70]	I^2 = 67%, Q = 79.12 (P < .001)
	Patel et al. 2024	0.60 [0.51-0.70]	I^2 = 68%, Q = 80.90 (P < .001)
	Janopaul-Naylor et al. 2024	0.58 [0.50-0.68]	I^2 = 65%, Q = 74.63 (P < .001)
	Huang S et al. 2024	0.58 [0.50-0.67]	I^2 = 62%, Q = 68.07 (P < .001)
	Hirata et al. 2024	0.59 [0.51-0.70]	I^2 = 68%, Q = 80.80 (P < .001)
	Catozzi et al. 2024 (NSCLC)	0.60 [0.51-0.70]	I^2 = 68%, Q = 81.02 (P < .001)
	Catozzi et al. 2024 (Other cancers)	0.60 [0.51-0.70]	I^2 = 68%, Q = 80.95 (P < .001)
	Yeung et al. 2023	0.61 [0.52-0.71]	I^2 = 67%, Q = 79.29 (P < .001)
	Rousseau et al. 2023	0.59 [0.50-0.70]	I^2 = 68%, Q = 80.85 (P < .001)
	Nomura et al. 2023	0.61 [0.52-0.71]	I^2 = 67%, Q = 78.68 (P < .001)
	Gonçalves et al. 2023	0.60 [0.51-0.71]	I^2 = 68%, Q = 80.05 (P < .001)
	Dizman et al. 2023	0.60 [0.51-0.70]	I^2 = 68%, Q = 81.02 (P < .001)
	Karaboué et al. 2022	0.62 [0.54-0.71]	I^2 = 63%, Q = 69.83 (P < .001)
	Qian et al. 2021	0.60 [0.51-0.71]	I^2 = 68%, Q = 80.20 (P < .001)
NSCLC	Huang Z et al. 2026	0.61 [0.49-0.78]	I^2 = 70%, Q = 26.40 (P < .001)
	Tsukaguchi et al. 2025	0.57 [0.44-0.74]	I^2 = 75%, Q = 32.17 (P < .001)
	McMillan et al. 2025	0.58 [0.45-0.75]	I^2 = 75%, Q = 32.38 (P < .001)
	Iwahashi et al. 2025	0.56 [0.43-0.73]	I^2 = 74%, Q = 31.18 (P < .001)
	Huang Z et al. 2025 Mar	0.60 [0.46-0.78]	I^2 = 69%, Q = 26.23 (P < .001)
	Gomez-Randulfe et al. 2025	0.55 [0.44-0.68]	I^2 = 59%, Q = 19.64 (P = .01)
	Hirata et al. 2024	0.57 [0.45-0.73]	I^2 = 75%, Q = 32.12 (P < .001)
	Catozzi et al. 2024 (NSCLC)	0.57 [0.44-0.75]	I^2 = 75%, Q = 32.36 (P < .001)
	Rousseau et al. 2023	0.57 [0.44-0.74]	I^2 = 75%, Q = 32.13 (P < .001)
	Karaboué et al. 2022	0.63 [0.52-0.76]	I^2 = 62%, Q = 21.31 (P = .01)
Melanoma	Nepote et al. 2026	0.58 [0.35-0.94]	I^2 = 70%, Q = 10.12 (P = .02)
	Amici et al. 2025	0.42 [0.30-0.60]	I^2 = 0%, Q = 0.89 (P = .83)
	Yeung et al. 2023	0.56 [0.31-1.01]	I^2 = 70%, Q = 9.96 (P = .02)
	Gonçalves et al. 2023	0.54 [0.30-0.99]	I^2 = 73%, Q = 11.02 (P = .01)
	Qian et al. 2021	0.54 [0.29-0.99]	I^2 = 73%, Q = 11.21 (P = .01)
Gastric cancer	Ishizuka et al. 2025	0.50 [0.27-0.89]	I^2 = 55%, Q = 2.22 (P = .14)
	Cheng et al. 2025	0.52 [0.27-0.99]	I^2 = 67%, Q = 3.06 (P = .08)
	Tanaka et al. 2024	0.66 [0.52-0.83]	I^2 = 0%, Q = 0.06 (P = .81)

Abbreviations: HR, hazard ratio; CI, confidence interval; NSCLC, non-small cell lung cancer

(B) Progression-free survival

Subgroup	Excluded study	HR [95% CI]	Heterogeneity
Overall	Nepote et al. 2026	0.63 [0.55-0.72]	I^2 = 68%, Q = 66.01 (P < .001)
	Huang Z et al. 2026	0.65 [0.57-0.74]	I^2 = 61%, Q = 53.64 (P < .001)
	Tsukaguchi et al. 2025	0.61 [0.53-0.71]	I^2 = 68%, Q = 66.34 (P < .001)
	Naganuma et al. 2025	0.61 [0.53-0.70]	I^2 = 66%, Q = 61.05 (P < .001)
	McMillan et al. 2025	0.62 [0.54-0.72]	I^2 = 69%, Q = 67.68 (P < .001)
	Iwahashi et al. 2025	0.61 [0.53-0.71]	I^2 = 69%, Q = 66.97 (P < .001)
	Ishizuka et al. 2025	0.61 [0.53-0.71]	I^2 = 69%, Q = 67.63 (P < .001)

© 2026 Inoue S et al. *JAMA Network Open*

	Huang Z et al. 2025 Dec	0.63 [0.55-0.73]	I^2 = 67%, Q = 63.27 (P < .001)
	Huang Z et al. 2025 Mar	0.63 [0.54-0.72]	I^2 = 66%, Q = 62.06 (P < .001)
	Gonçalves et al. 2025	0.63 [0.54-0.72]	I^2 = 69%, Q = 66.81 (P < .001)
	Gomez-Randulfe et al. 2025	0.60 [0.53-0.70]	I^2 = 64%, Q = 58.47 (P < .001)
	Cheng et al. 2025	0.61 [0.53-0.71]	I^2 = 69%, Q = 67.46 (P < .001)
	Amici et al. 2025	0.61 [0.53-0.70]	I^2 = 66%, Q = 61.55 (P < .001)
	Zheng et al. 2024	0.62 [0.54-0.72]	I^2 = 69%, Q = 66.71 (P < .001)
	Tanaka et al. 2024	0.63 [0.55-0.73]	I^2 = 67%, Q = 63.78 (P < .001)
	Ruiz-Torres et al. 2024	0.62 [0.53-0.72]	I^2 = 69%, Q = 67.72 (P < .001)
	Patel et al. 2024	0.62 [0.53-0.71]	I^2 = 69%, Q = 67.69 (P < .001)
	Janopaul-Naylor et al. 2024	0.61 [0.53-0.70]	I^2 = 67%, Q = 63.78 (P < .001)
	Hirata et al. 2024	0.63 [0.54-0.72]	I^2 = 68%, Q = 66.23 (P < .001)
	Yeung et al. 2023	0.63 [0.54-0.72]	I^2 = 69%, Q = 66.69 (P < .001)
	Rousseau et al. 2023	0.62 [0.53-0.71]	I^2 = 69%, Q = 67.74 (P < .001)
	Nomura et al. 2023	0.63 [0.55-0.73]	I^2 = 68%, Q = 64.86 (P < .001)
	Karaboué et al. 2022	0.63 [0.55-0.73]	I^2 = 66%, Q = 62.49 (P < .001)
NSCLC	Huang Z et al. 2026	0.66 [0.53-0.81]	I^2 = 68%, Q = 22.21 (P = .002)
	Tsukaguchi et al. 2025	0.57 [0.43-0.75]	I^2 = 79%, Q = 32.82 (P < .001)
	McMillan et al. 2025	0.59 [0.45-0.78]	I^2 = 80%, Q = 35.12 (P < .001)
	Iwahashi et al. 2025	0.57 [0.43-0.76]	I^2 = 79%, Q = 33.71 (P < .001)
	Huang Z et al. 2025 Mar	0.60 [0.45-0.80]	I^2 = 77%, Q = 30.68 (P < .001)
	Gomez-Randulfe et al. 2025	0.56 [0.43-0.71]	I^2 = 67%, Q = 21.32 (P = .003)
	Hirata et al. 2024	0.61 [0.47-0.79]	I^2 = 79%, Q = 33.84 (P < .001)
	Rousseau et al. 2023	0.58 [0.43-0.77]	I^2 = 80%, Q = 34.93 (P < .001)
	Karaboué et al. 2022	0.63 [0.50-0.80]	I^2 = 77%, Q = 30.30 (P < .001)
Melanoma	Nepote et al. 2026	0.73 [0.37-1.46]	I^2 = 79%, Q = 4.72 (P = .03)
	Amici et al. 2025	0.46 [0.29-0.73]	I^2 = 0%, Q = 0.41 (P = .52)
	Yeung et al. 2023	0.65 [0.24-1.82]	I^2 = 77%, Q = 4.27 (P = .04)
Gastric cancer	Ishizuka et al. 2025	0.54 [0.26-1.10]	I^2 = 74%, Q = 3.78 (P = .05)
	Cheng et al. 2025	0.53 [0.28-1.01]	I^2 = 74%, Q = 3.85 (P = .05)
	Tanaka et al. 2024	0.71 [0.57-0.90]	I^2 = 0%, Q = 0.07 (P = .79)

Abbreviations: HR, hazard ratio; CI, confidence interval; NSCLC, non-small cell lung cancer

© 2026 Inoue S et al. *JAMA Network Open*

eFigure 1. Risk of bias assessment according to ROBINS-I tool

Abbreviations: ROBINS-I, Risk Of Bias In Non-randomized Studies - of Interventions

© 2026 Inoue S et al. *JAMA Network Open*

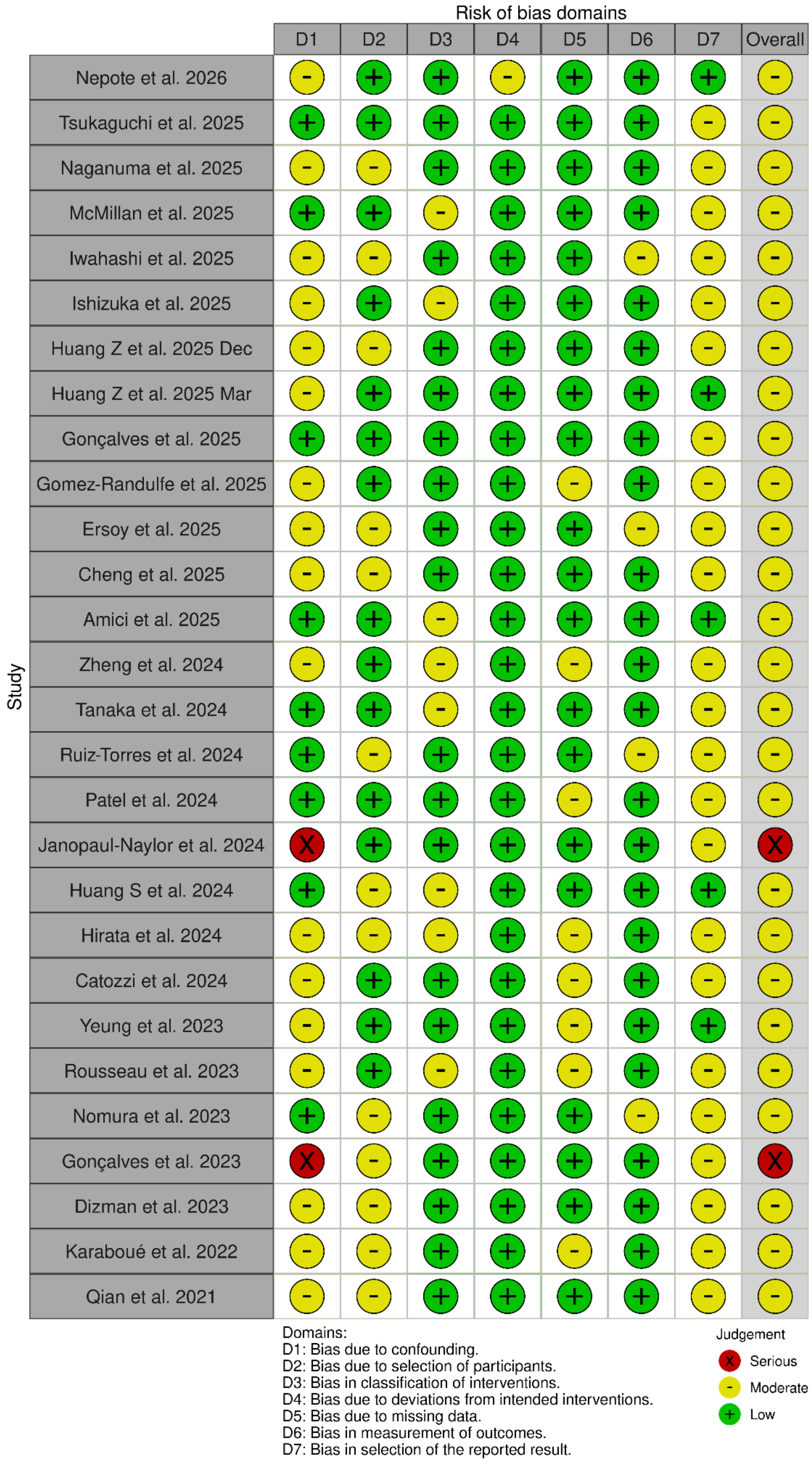

© 2026 Inoue S et al. *JAMA Network Open*

eFigure 2. Risk of bias assessment according to RoB 2 tool

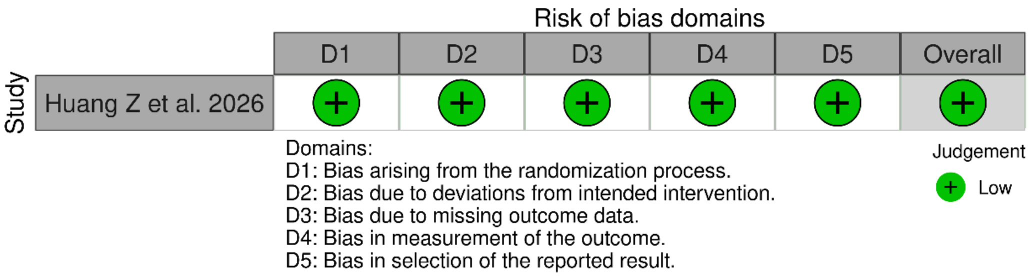

Abbreviations: RoB, Risk of Bias

© 2026 Inoue S et al. *JAMA Network Open*

eFigure 3. Funnel plots assessing publication bias

(A) Overall survival (Egger's test p-value = 0.28)

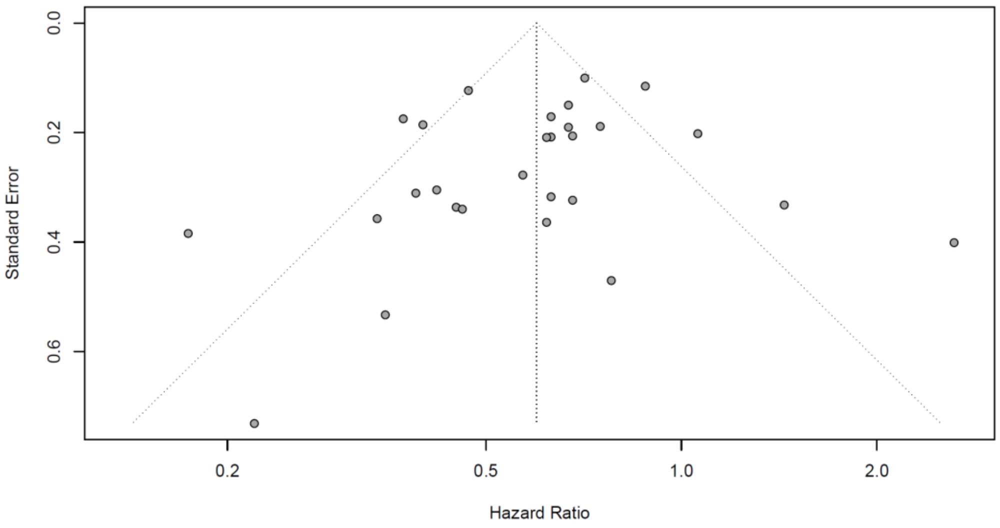

(B) Progression-free survival (Egger's test p-value = 0.06)

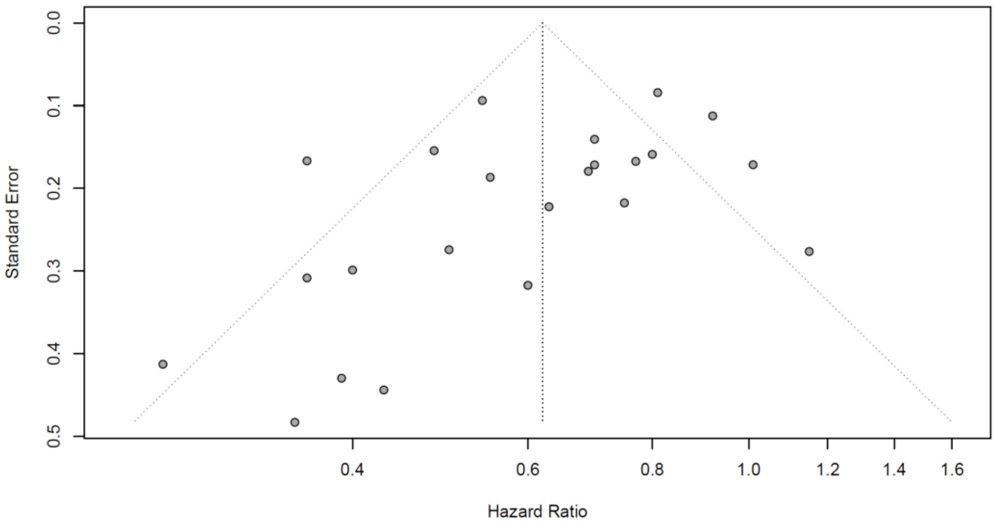

© 2026 Inoue S et al. *JAMA Network Open*

eFigure 4. Sensitivity meta-analysis using only adjusted hazard ratios for overall survival

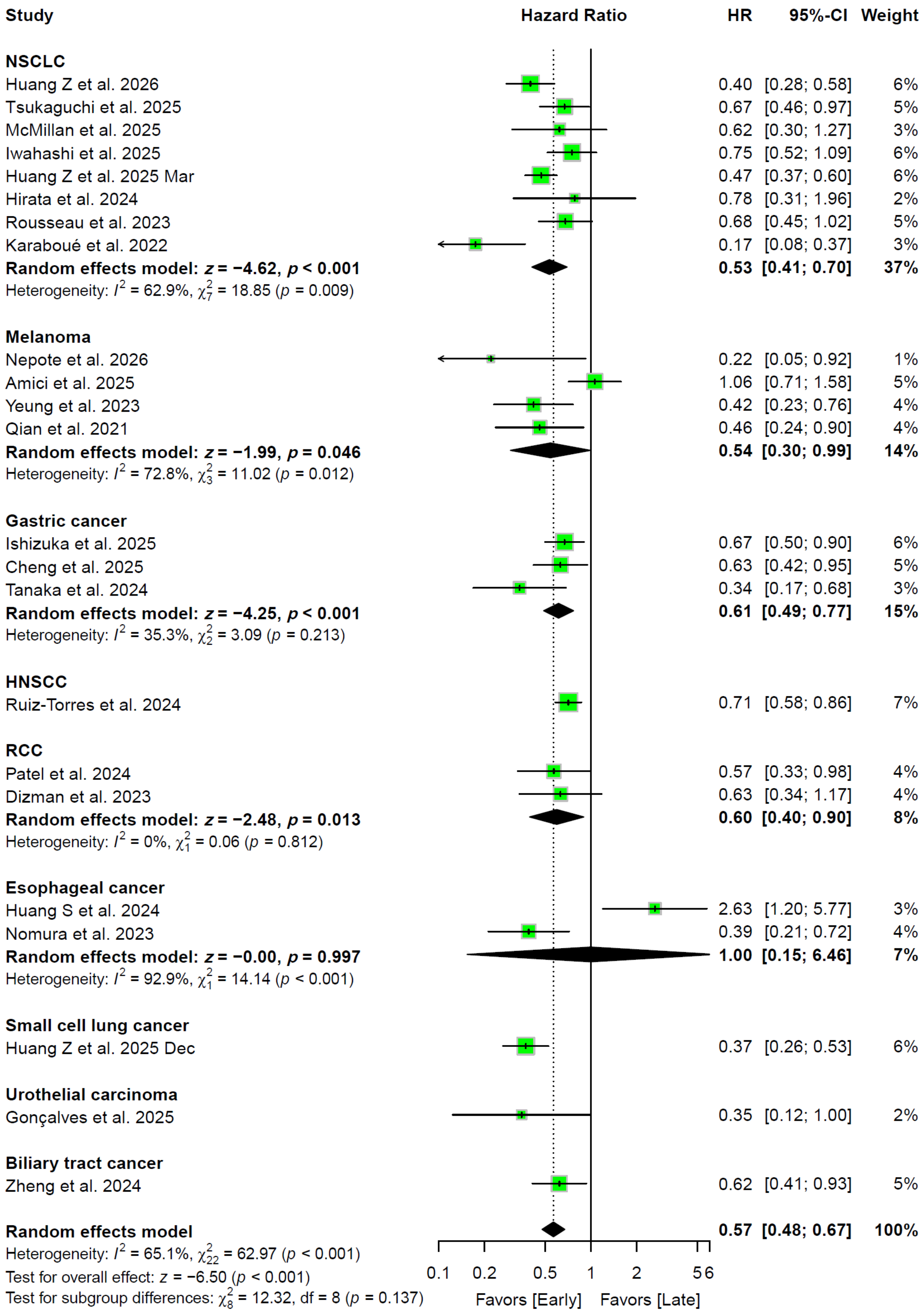

© 2026 Inoue S et al. *JAMA Network Open*

Abbreviations: HR, hazard ratio; CI, confidence interval; NSCLC, non-small cell lung cancer; HNSCC, head and neck squamous cell carcinoma; RCC, renal cell carcinoma

© 2026 Inoue S et al. *JAMA Network Open*

eFigure 5. Sensitivity meta-analysis using only unadjusted hazard ratios for overall survival

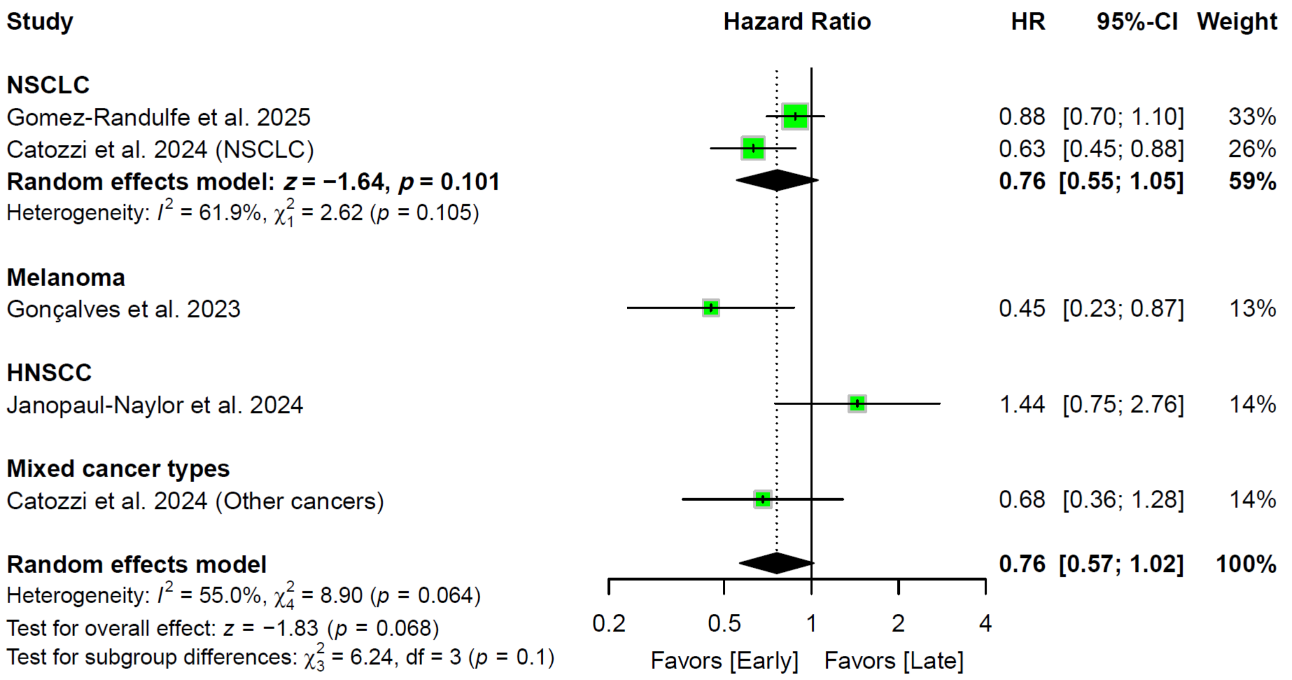

Abbreviations: HR, hazard ratio; CI, confidence interval; NSCLC, non-small cell lung cancer; HNSCC, head and neck squamous cell carcinoma

© 2026 Inoue S et al. *JAMA Network Open*

eFigure 6. Sensitivity meta-analysis using only adjusted hazard ratios for progression-free survival

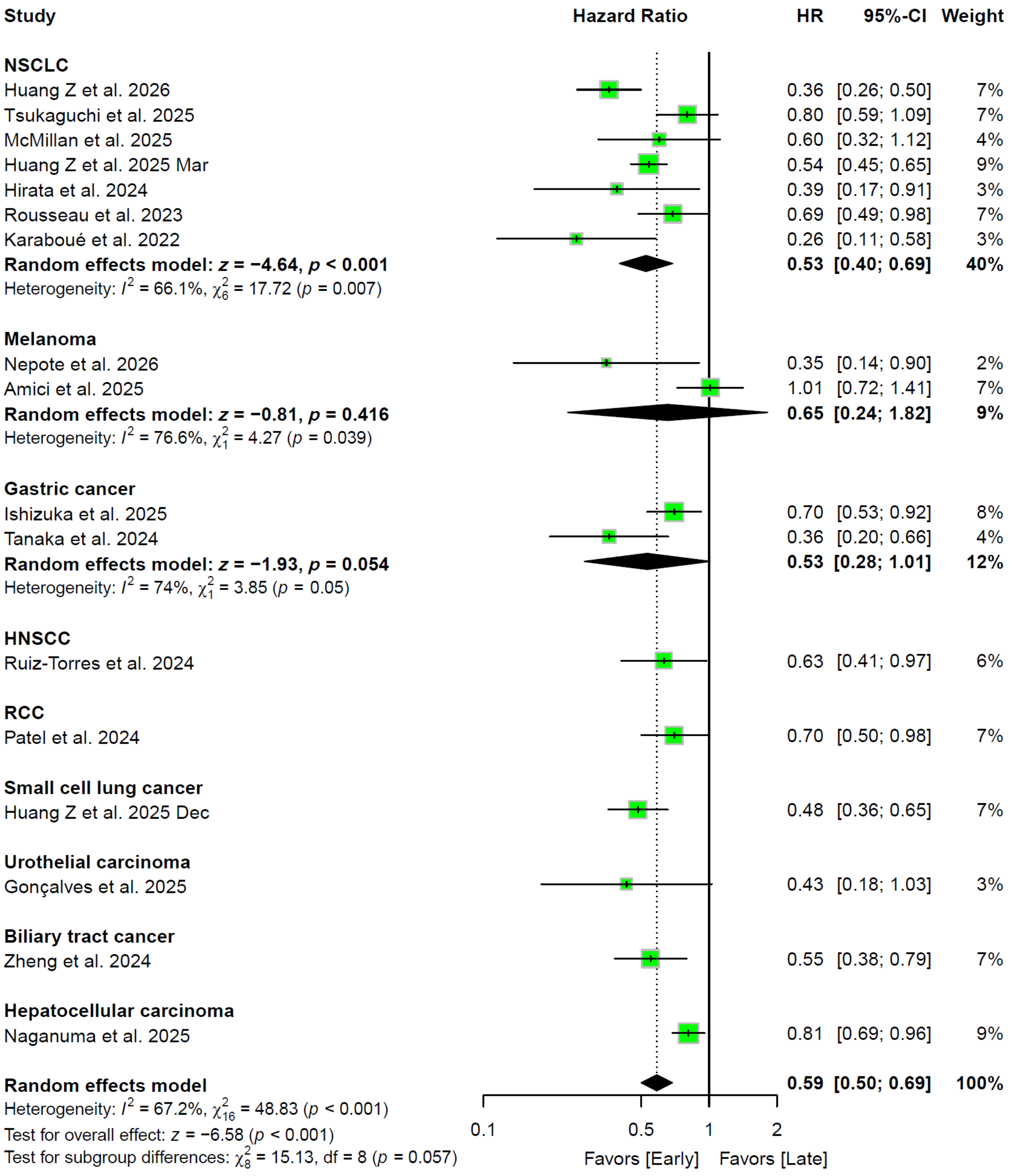

Abbreviations: HR, hazard ratio; CI, confidence interval; NSCLC, non-small cell lung cancer; HNSCC, head and neck squamous cell carcinoma; RCC, renal cell carcinoma

© 2026 Inoue S et al. *JAMA Network Open*

eFigure 7. Sensitivity meta-analysis using only unadjusted hazard ratios for progression-free survival

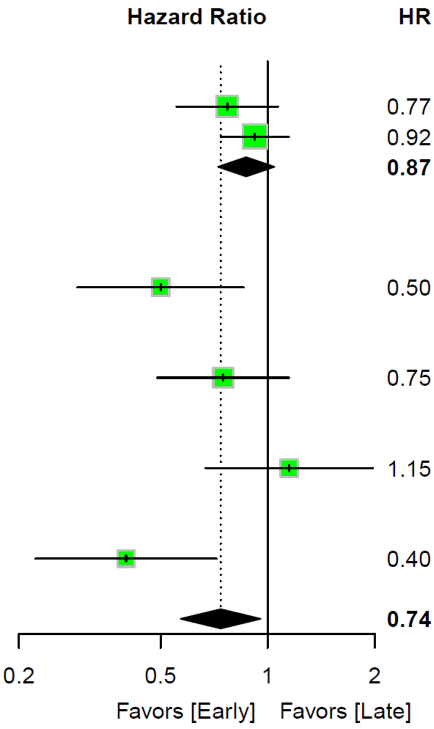

Study	HR	95%-CI	Weight
**NSCLC**			
Iwahashi et al. 2025	0.77	[0.55; 1.07]	20%
Gomez-Randulfe et al. 2025	0.92	[0.74; 1.15]	25%
**Random effects model: $z = -1.49$, $p = 0.137$**	**0.87**	**[0.72; 1.05]**	**45%**
Heterogeneity: $I^2 = 0\%$, $\chi^2_1 = 0.78$ ($p = 0.377$)			
**Melanoma**			
Yeung et al. 2023	0.50	[0.29; 0.86]	13%
**Gastric cancer**			
Cheng et al. 2025	0.75	[0.49; 1.15]	17%
**HNSCC**			
Janopaul-Naylor et al. 2024	1.15	[0.67; 1.98]	13%
**Esophageal cancer**			
Nomura et al. 2023	0.40	[0.22; 0.72]	12%
**Random effects model**	**0.74**	**[0.57; 0.96]**	**100%**

Heterogeneity: $I^2 = 57.3\%$, $\chi^2_5 = 11.72$ ($p = 0.039$)
Test for overall effect: $z = -2.30$ ($p = 0.022$)
Test for subgroup differences: $\chi^2_4 = 10.94$, df = 4 ($p = 0.027$)

Abbreviations: HR, hazard ratio; CI, confidence interval; NSCLC, non-small cell lung cancer; HNSCC, head and neck squamous cell carcinoma

© 2026 Inoue S et al. *JAMA Network Open*

eFigure 8. Exploratory subgroup analysis by definitions of early versus late time of day

(A) Overall survival

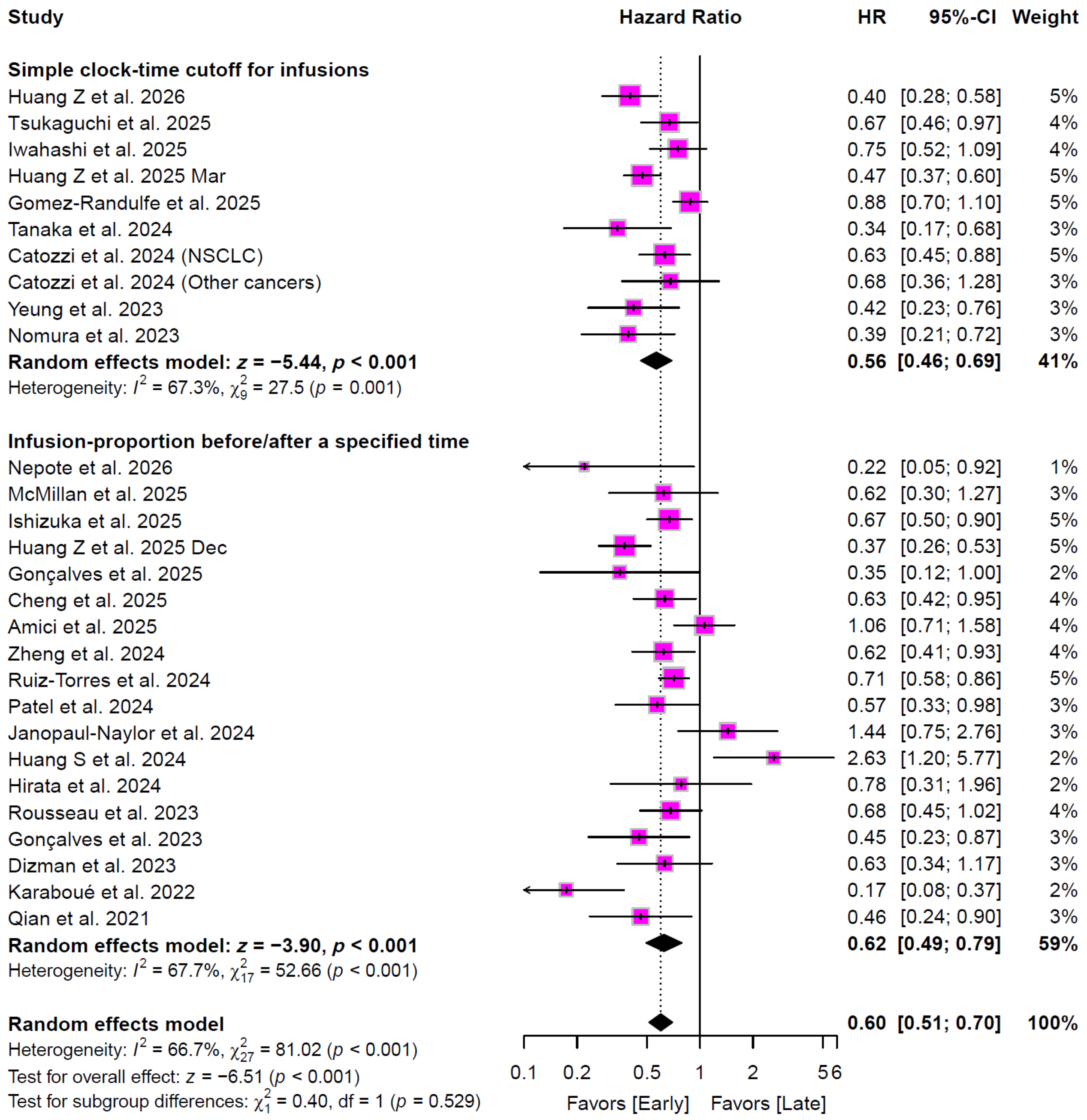

© 2026 Inoue S et al. *JAMA Network Open*

(B) Progression-free survival

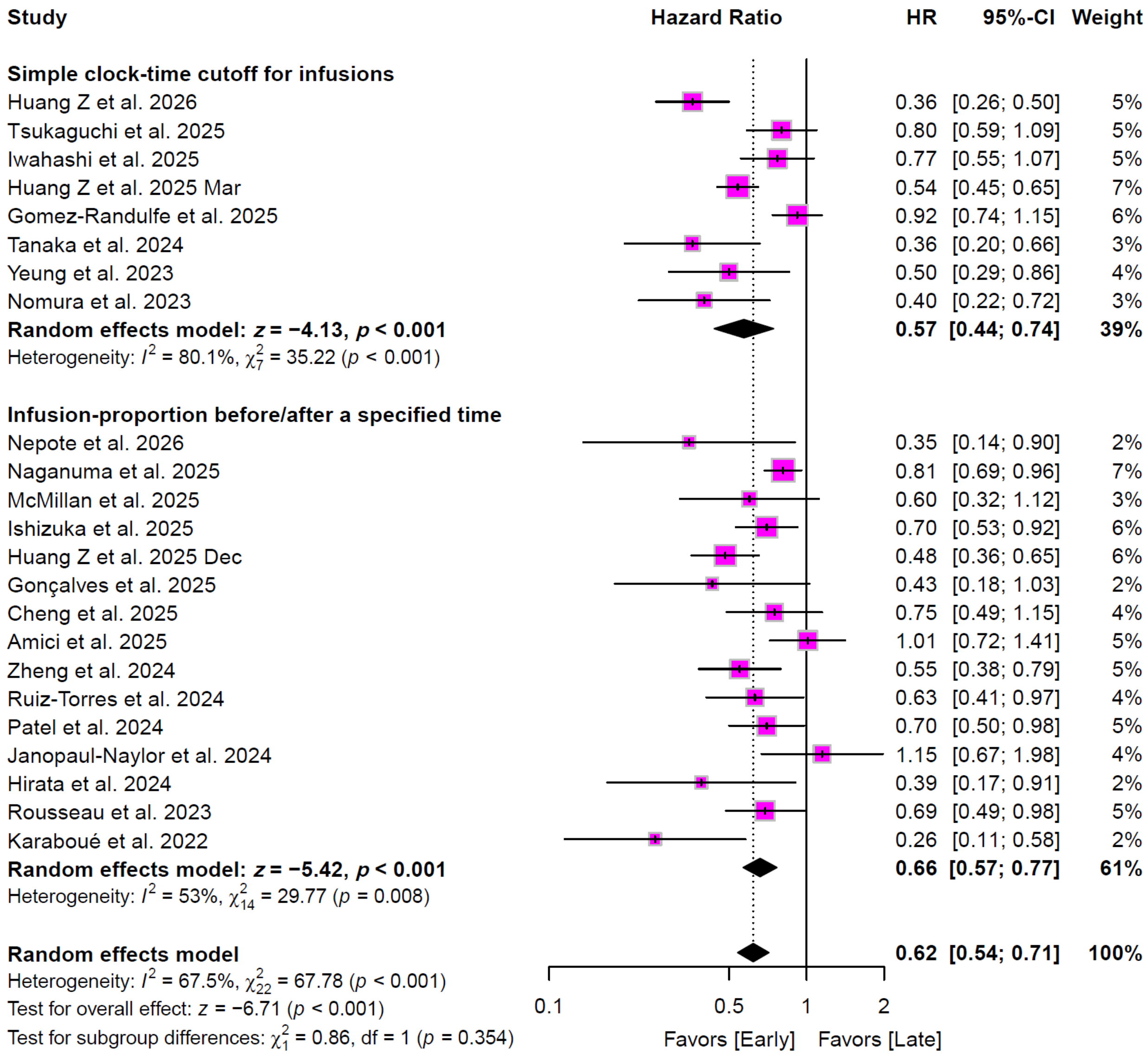

Abbreviations: HR, hazard ratio; CI, confidence interval

© 2026 Inoue S et al. *JAMA Network Open*

eFigure 9. Exploratory subgroup analysis by ICI regimen type

(A) Overall survival

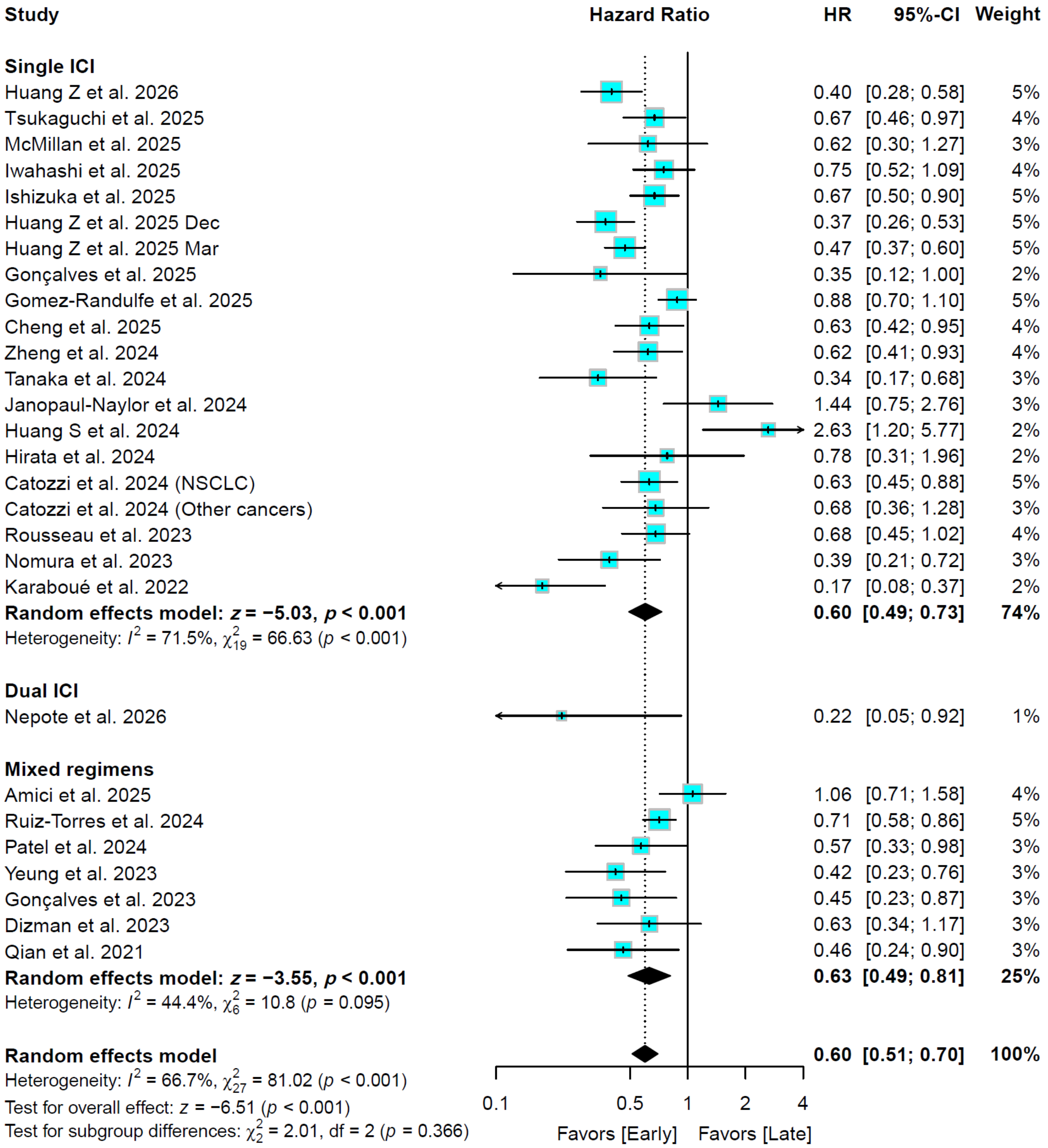

© 2026 Inoue S et al. *JAMA Network Open*

(B) Progression-free survival

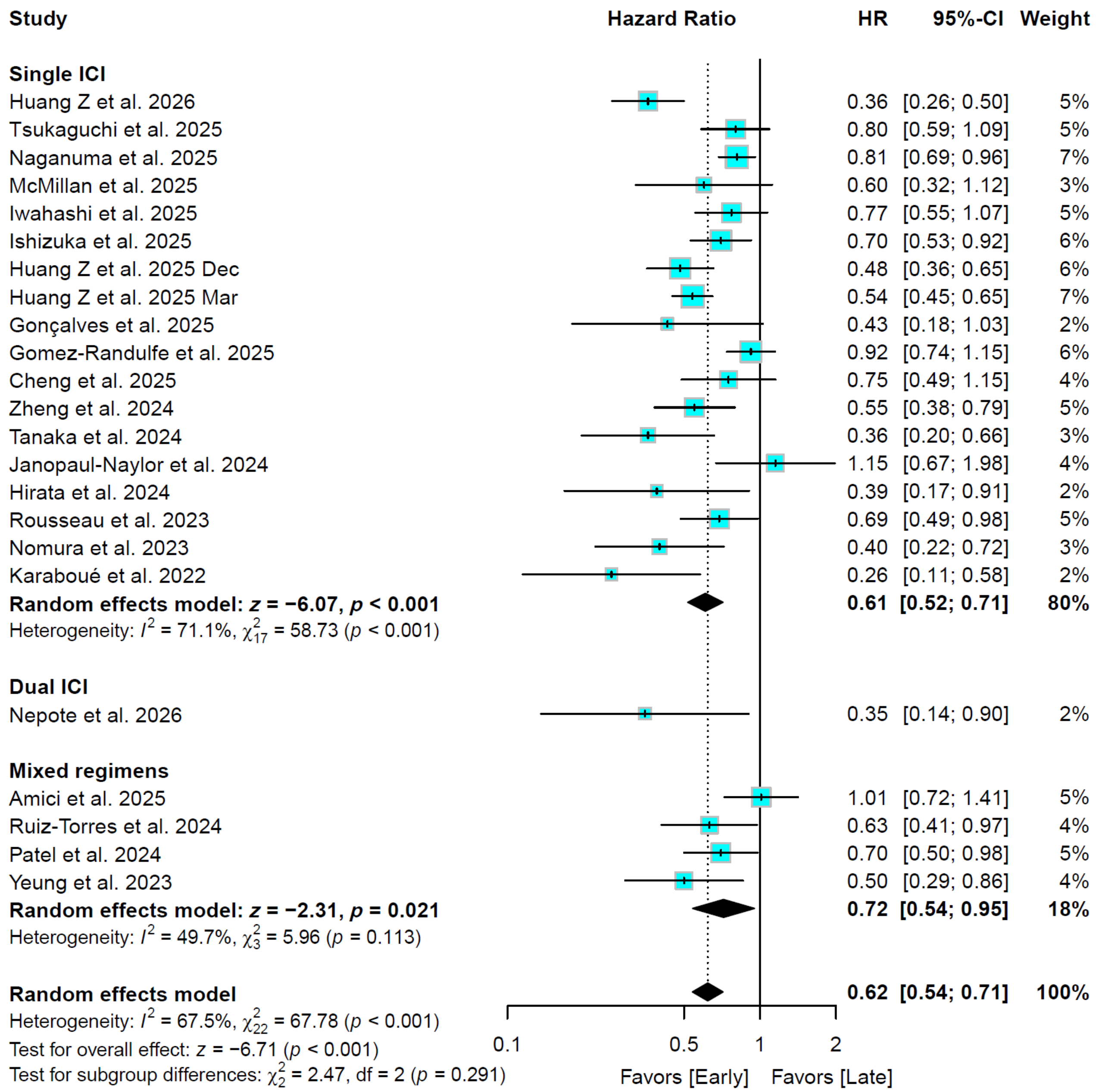

Abbreviations: HR, hazard ratio; CI, confidence interval; ICI, immune checkpoint inhibitor

© 2026 Inoue S et al. *JAMA Network Open*
