# Supplementary material for: Time-of-Day Immunotherapy Administration and Outcomes in Advanced Cancers: A Systematic Review and Meta-Analysis
Source: JAMA Netw Open. 2026 May 5;9(5):e2610815. doi: 10.1001/jamanetworkopen.2026.10815 (PMC13147196; doi:10.1001/jamanetworkopen.2026.10815)
Supplement: Supplement 2. — Data Sharing Statement [file jamanetwopen-e2610815-s002.pdf]

Data Sharing Statement

Inoue. Time-of-Day Immunotherapy Administration and Outcomes in Advanced Cancers. *JAMA Netw Open*. Published May 05, 2026. doi:10.1001/jamanetworkopen.2026.10815

Data
Data available: No
